# New insight into flavivirus maturation from structure/function studies of the yellow fever virus envelope protein complex

**DOI:** 10.1128/mbio.00706-23

**Published:** 2023-08-21

**Authors:** E. Crampon, E. Covernton, M. C. Vaney, M. Dellarole, S. Sommer, A. Sharma, A. Haouz, P. England, J. Lepault, S. Duquerroy, F. A. Rey, G. Barba-Spaeth

**Affiliations:** 1 Institut Pasteur, Université Paris Cité, CNRS UMR 3569, Unité de Virologie Structurale, Paris, France; 2 Institut Pasteur, Université Paris Cité, CNRS UMR 3528, Plateforme de Cristallographie-C2RT, Paris, France; 3 Institut Pasteur, Université Paris Cité, CNRS UMR 3528, Plateforme de Biophysique Moléculaire-C2RT, Paris, France; 4 Institute for Integrative Biology of the Cell (I2BC), CEA, CNRS, Université Paris-Saclay, Gif-sur-Yvette, France; 5 Université Paris-Saclay, Faculté des Sciences, Orsay, France; Columbia University Medical College, New York, New York, USA

**Keywords:** flavivirus maturation, X-ray crystallography, viral fusion

## Abstract

Flavivirus particle maturation, a process essential for virus infectivity, has been mostly studied using dengue virus. In infected cells, immature icosahedral virions bud into the endoplasmic reticulum. Budding is induced by lateral contacts between heterodimers of transmembrane glycoproteins prM and E. During exocytosis through the trans-Golgi network (TGN), the acidic environment triggers a major particle reorganization in which E forms head-to-tail dimers and furin cleaves prM into globular pr and transmembrane M proteins. pr remains bound to E at acidic pH and blocks its fusogenic activity, but at neutral pH, its affinity for E drops and pr is shed from the particle in the extracellular environment, leaving a virion activated for fusion at low pH. We report here that recombinant yellow fever virus (YFV) pr retains high affinity for soluble E (sE) at neutral pH—significantly shifting the current paradigm. The X-ray structure of the YFV pr/sE complex shows essentially the same pattern of interactions reported for dengue virus, while the X-ray structure of YFV sE at neutral pH shows the same canonical head-to-tail sE dimer. pr binding to the sE dimer is precluded by the E “150-loop,” indicating it must adopt a different conformation in the E dimers on virions at acidic pH for pr binding. We had previously reported a similar local reorganization of the E 150-loop at acidic pH for the tick-borne encephalitis virus, with the important difference that pr stabilized a soluble sE head-to-tail dimer, which is not the case for YFV.

**IMPORTANCE:**

All enveloped viruses enter cells by fusing their envelope with a target cell membrane while avoiding premature fusion with membranes of the producer cell—the latter being particularly important for viruses that bud at internal membranes. Flaviviruses bud in the endoplasmic reticulum, are transported through the TGN to reach the external milieu, and enter other cells via receptor-mediated endocytosis. The trigger for membrane fusion is the acidic environment of early endosomes, which has a similar pH to the TGN of the producer cell. The viral particles therefore become activated to react to mildly acidic pH only after their release into the neutral pH extracellular environment. Our study shows that for yellow fever virus (YFV), the mechanism of activation involves actively knocking out the fusion brake (protein pr) through a localized conformational change of the envelope protein upon exposure to the neutral pH external environment. Our study has important implications for understanding the molecular mechanism of flavivirus fusion activation in general and points to an alternative way of interfering with this process as an antiviral treatment.

## INTRODUCTION

The mosquito-borne yellow fever virus (YFV) is prototypic of the *Flavivirus* genus, which also includes several human pathogenic viruses such as dengue virus (DENV), Zika virus (ZIKV), Japanese encephalitis virus (JEV), and tick-borne encephalitis virus (TBEV) (1). YFV is endemic in sub-Saharan Africa and the Amazonian basin in South America, where it is maintained in sylvatic cycles of transmission among nonhuman primates (NHPs) via sylvatic mosquito species and sporadic urban cycles among humans involving *Aedes* mosquitoes (2, 3). An efficient vaccine is in use against yellow fever disease, yet the recent variants responsible for outbreaks in Brazil appear less sensitive to the antibodies produced by vaccination (4), raising concern about potential future outbreaks for which the vaccine could confer reduced protection.

Like all enveloped viruses, flaviviruses enter cells by fusing the viral envelope with a cellular membrane. This process is catalyzed by a dedicated membrane fusion protein present at the particle surface. Flaviviruses, which enter cells via receptor-mediated endocytosis, use protein E for this essential function. In infectious particles, E is primed to react to the mildly acidic pH of an endosome to undergo a fusogenic conformational change that drives the membrane fusion reaction. This process involves an initial insertion of E monomers into the endosomal membrane via a “fusion loop” at the viral-membrane-distal end of E, followed by E trimerization and reorganization of the E protomers into a hairpin that forces the two membranes against each other.

In the producer cells, flavivirus particles assemble at the membrane of the endoplasmic reticulum (ER), and the resulting particles are then transported to the cell surface through the trans-Golgi network (TGN). Like in early endosomes, the environment in the TGN is mildly acidic. A specific particle maturation process is therefore required to ensure that E activation for fusion at low pH occurs only after the particle has reached the external milieu of the producer cell. Here, we analyze the YFV particle maturation process to yield infectious virions, identifying YFV-specific features that shift the conceptual understanding of this process as derived from studies on other flaviviruses.

The flavivirus immature particles contain transmembrane glycoproteins prM and E in an envelope enclosing the viral genome as a ribonucleoprotein complex with the capsid (C) protein. Glycoprotein prM has been shown to play a chaperone role for folding of E into a functional membrane fusogen (5). During biosynthesis, the two proteins associate co-translationally in the ER to form a prM/E heterodimer. Lateral interactions between these heterodimers result in an intertwined assembly of head-to-head (prM/E)_3_ trimers that drive budding by inducing local curvature at the ER membrane to form closed, spiky immature particles with icosahedral symmetry (6 – 10). Pioneering structure-function studies with TBEV (11, 12) and dengue virus serotype 2 (DENV2) (13 – 15) have shown that exposure to the mildly acidic pH of the TGN during exocytosis results in dissociation of (prM/E)_3_ trimers, which reorganize into (prM/E)_2_ dimers to make a smooth particle in which prM exposes a cleavage site for furin, a TGN-resident cellular protease. Furin then cleaves prM into a globular, N-terminal “pr” protein and an extended, C-terminally membrane-anchored “M” protein that lies below the E protein dimers. These and subsequent studies (16, 17) further showed that pr bound to E blocks insertion of the E fusion loop into membranes. They also showed that pr’s affinity for E is high at acidic pH but drops to undetectable levels at neutral pH. Because the pH in the extracellular milieu is neutral, pr was initially proposed to be passively “shed” from the virion as soon as it exits the cell, leaving an activated particle ready to react to the mildly acidic pH of an endosome upon endocytic uptake by a target cell.

The organization of the immature particles at low pH is the same as that of mature particles, with 90 head-to-tail E dimers forming a “herringbone” pattern (18, 19). Protein E is a prototypic class II fusion protein (20), with three β-sheet-rich domains termed DI, DII, and DIII. The fusion loop is located at the tip of DII and is partially buried at the E dimer interface at the site of pr/E contacts (14). Recent studies with TBEV have shown that pr binding to the E dimer is mediated by a localized conformational change of E involving a loop in DI—termed the “150-loop”—which at neutral pH packs against the fusion loop across the E dimer interface. This loop pops open at acidic pH, creating a pocket where pr binds and stabilizes the interface. When the virion is exposed to neutral pH, the 150-loop closes in and knocks out pr, which is not passively released by an affinity drop as concluded earlier, but by the 150-loop acting as a snap-lock that relays pr in helping mask the fusion loop at neutral pH (21).

We show here that for YFV, the pr affinity for the E monomer remains high even at neutral pH, contrary to the observations made in the case of DENV2 and TBEV. The X-ray structure of YFV pr in complex with soluble E (sE) displays a similar number of polar interactions as in the previously described structures of its counterpart in DENV and TBEV. Yet unlike TBEV, pr does not stabilize the sE dimer at acidic pH. In the absence of pr, YFV sE remains monomeric at neutral pH but crystallizes as a head-to-tail dimer, as observed at the surface of flavivirus mature particles. Our biochemical experiments further showed that, similar to its counterparts in other flaviviruses, pr blocks insertion of YFV sE into liposomes, except that a 1:1 stoichiometry is enough for a complete effect, in contrast to the 10-fold pr excess required for DENV2 (16). Yet, we found that a 10-fold pr excess was required to block the fusion of YFV virions with liposomes at low pH. This result indicated that the kinetics of this process is important, such that pr cannot directly access its binding site and requires prior E dimer dissociation through the effect of low pH. To block membrane fusion, a sufficiently high pr excess is thus required for it to find its binding site before the fusion loops have been inserted into the liposomes. The structure of the YFV sE dimer also showed that the conformation of the 150-loop interferes with pr binding, again pointing to a key role of this loop in pr ejection and particle activation. Our results illustrate alternative ways in which the 150-loop participates in the flavivirus activation process depending on the flavivirus species.

## RESULTS

### Structure of the YFV pr/sE complex

**Fig 1:**
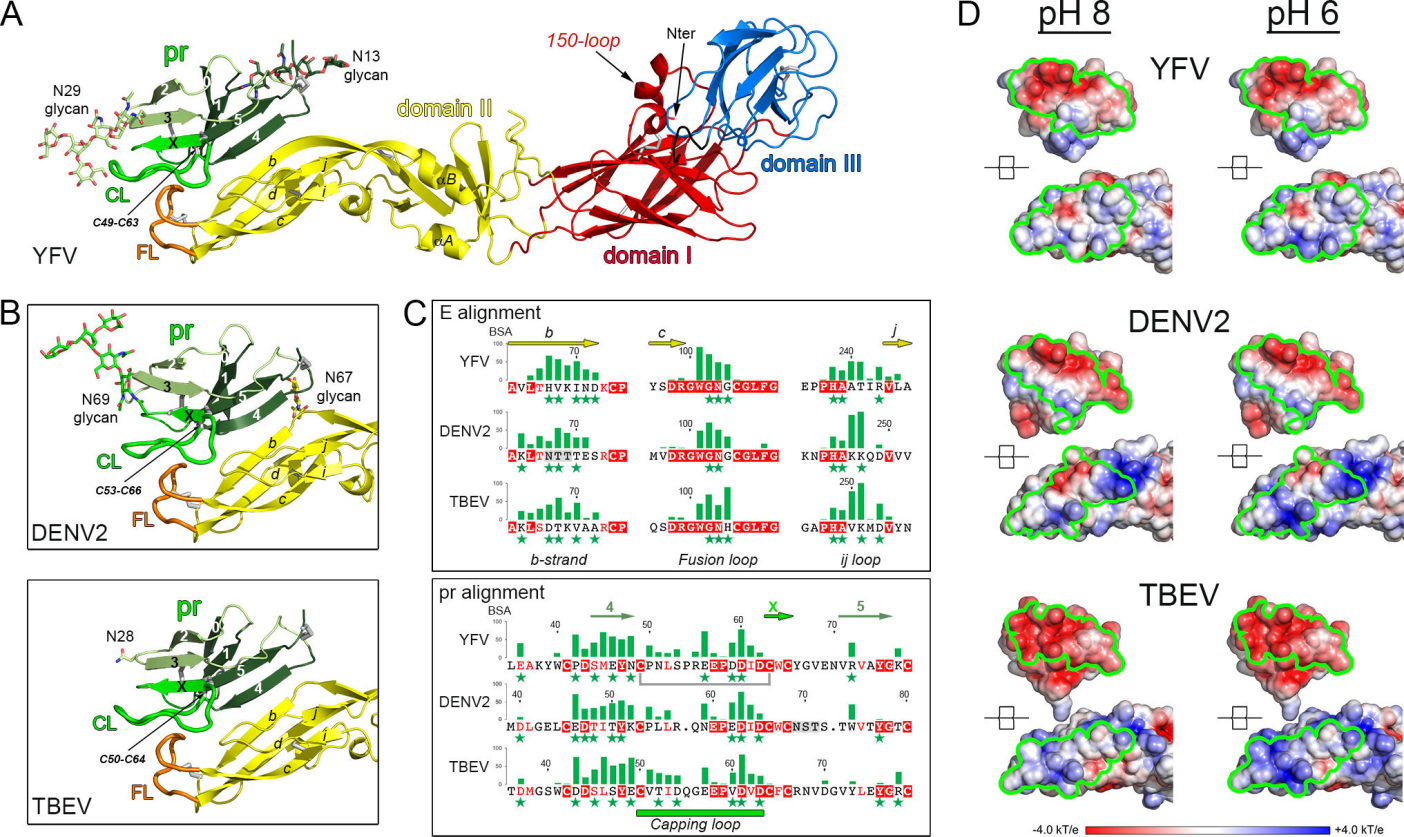
Structure of the YFV pr/sE complex and comparison to other flaviviruses. (**A**) Structure of the YFV pr/sE complex. E is colored according to the classical flavivirus E domains DI, DII, and DIII in red, yellow, and blue, respectively. The fusion loop (FL) is in orange. pr is in various shades of green and is glycosylated in positions Asn13 and Asn29 as indicated. The pr capping loop (CL) is displayed in bright green. The black arrows point the 150-loop, the N-terminal residue, and the disulfide bond Cys49-Cys63 that stabilizes the CL. (**B**) Closeup of the pr/sE interaction in DENV2 (PDB 3C5X) (top panel) and in TBEV (PDB 7QRE) (bottom panel). The views are in the same orientation as in (**A**). The positions of glycans are represented by sticks. The conserved disulfide bonds stabilizing the CL in pr are indicated in both panels. In DENV2, pr is glycosylated in position N69, and the glycan N67 on E is indicated. In TBEV, the position N28 on pr indicates the glycan, which is not visible in the structure. (**C**) Histograms of the van der Waals contacts between pr and E. E (top) and pr (bottom) structural alignments for YFV, DENV2, and TBEV. The conserved residues between the three viruses are displayed in white on red background and similar residues are in red. The gray background in DENV2 sequences indicates the positions of the glycans. Only sections of E that interact with pr are displayed (*b*-strand, fusion loop, and *ij* loop). The green stars below residues indicate polar interaction between pr and E. In YFV, gray bracket lines underline the two residues Cys49 and Cys63 forming a disulfide bond. (**D**) Open-book representation of the electrostatic surface potential on pr and E of pr/sE complexes. The surfaces are colored according to the scale bar from −4 kT/e (red) to +4.0 kT/e (blue) and computed at pH 8 (left panel) and at pH 6 (right panel). The interaction surfaces of pr/sE are outlined in green. The potential of pr (top views) is not dependent on the pH while the potential of E (bottom views) appears to direct the change of the electrostatic at the interaction surface with pr.

We produced the soluble YFV E ectodomain (sE) in insect S2 cells with a construct expressing the prM-E region of the viral polyprotein, with a strep-tag and stop codon engineered immediately after DIII, as done previously for production of sE from other flaviruses (22). In this system, prM is cleaved into pr and M by furin in the transfected cell. M remains membrane-anchored in the producer cell while the remaining pr/sE complex is secreted. For most flaviviruses, pr dissociates from sE upon reaching the extracellular milieu at neutral pH. In the case of YFV instead, this procedure led to the purification of a stable pr/sE complex at pH 8. In the case of DENV2, determination of the structure of the pr/sE complex required engineering a soluble linker bypassing the TM segment of prM (13) to make a covalent complex that could be purified at neutral pH. We instead obtained crystals at pH 8 of the YFV noncovalently linked pr/sE complex (see supplementary material and methods section) that yielded a structure to 2.7 Å resolution with a free R factor of 19% (Fig. 1A; Table S1). The structure showed that pr binds to the tip of E DII in the same way as shown in the DENV2 covalently linked complex (13) and in the intra-protomer contacts of the TBEV (pr/sE)_2_ dimer at acidic pH (Fig. 1B), in which no artificial linker was required (21).

YFV E has no N-glycosylation sequons and YFV pr has two, with attached glycans at positions Asn13 and Asn29, at opposite ends of the elongated pr molecule, as clearly observed in the electron density (Fig. 1A). The capping loop in pr wraps around the sE fusion loop (FL, shown in orange in Fig. 1A) and is stabilized by a disulfide bond between Cys49 and Cys63 (YFV numbering) (Fig. 1A), which are strictly conserved across human pathogenic flaviviruses (Fig. 1C, bottom panel). The association of the YFV pr/E complex is thus supported by interactions between residues that are conserved across flaviviruses (highlighted by a green background in Table S2) and nonconserved residues, but which amount to a similar set of interactions (Fig. 1C; Table S2). In summary, YFV pr makes multiple polar and van der Waals interactions with E, in particular with E β-strand *b* and the fusion loop, in a pattern very similar to that described for DENV2 and TBEV (Fig. 1; Tables S2 and S3). A comparison of the surface electrostatic potential of E and pr surfaces at their binding sites at pH 8 and 6 (Fig. 1D, top panel) (calculated on the structure obtained at pH 8) indicated charge complementarity between the interacting surfaces, which became more marked at pH 6, suggesting higher affinity upon partial protonation. A similar comparison of the interacting surfaces in the DENV2 and TBEV pr/sE complexes showed a similar trend (Fig. 1D).

### The YFV sE dimer

The sE dimer structure, determined by molecular replacement and refined to a free R factor of ~27% (Table S1, see Materials and Methods), showed the same head-to-tail dimer conformation observed in the previously reported structure of YFV 17D sE dimer in complex with the antigen-binding fragment of the monoclonal antibody A5 (23). The overall structure matches that of the flavivirus head-to-tail E dimers at the surface of flavivirus particles (18, 19). The YFV sE dimer organization will be discussed in more detail in a subsequent section.

### Chaperone role of pr

**Fig 2:**
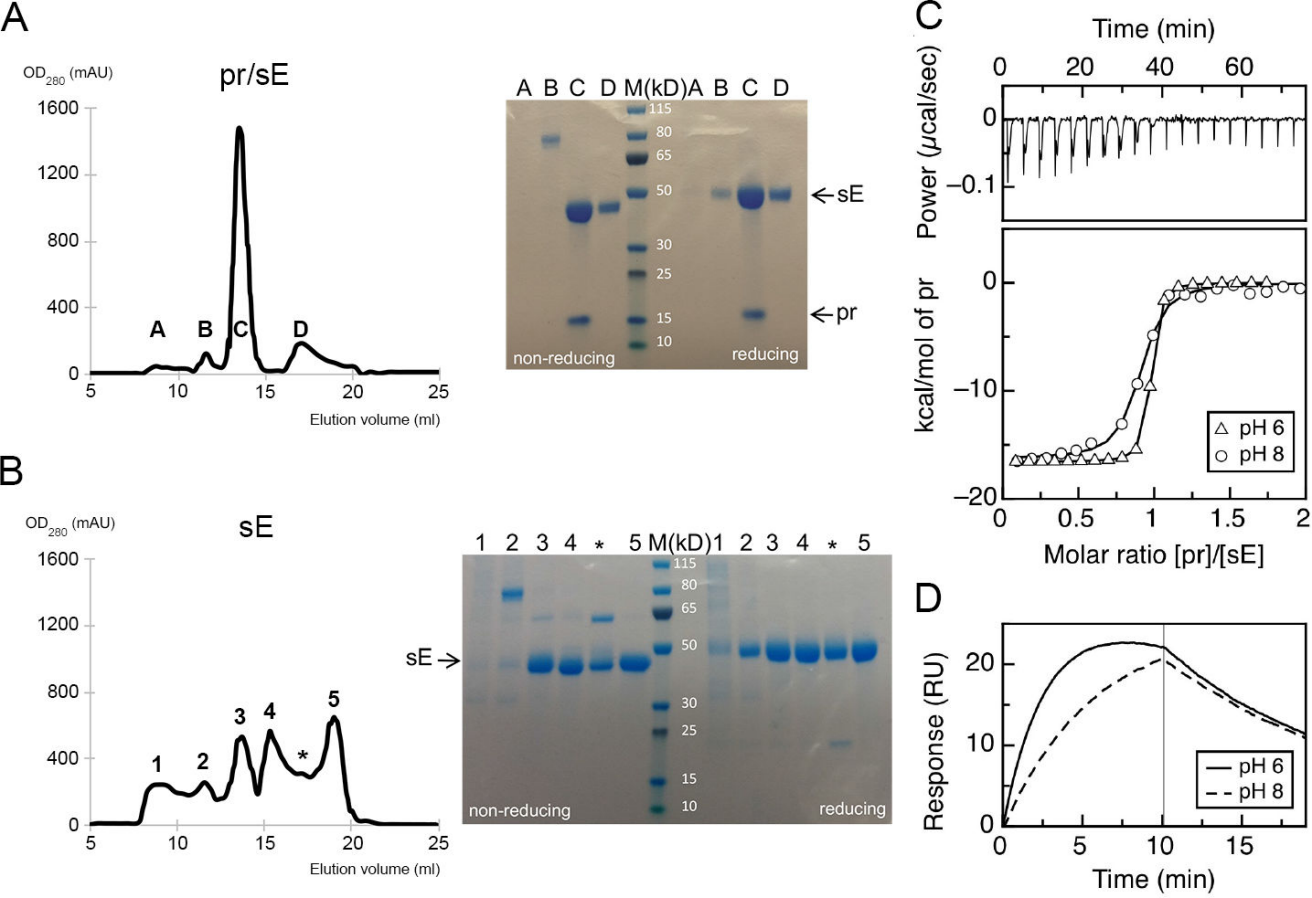
Biophysical characterizations of the pr/sE interaction and chaperone activity of prM on the E protein. (**A and B**) Left panels: SEC profiles of YFV sE expressed with prM (**A**), showing four peaks, labeled A, B, C, and D, and of sE expressed without prM (**B**), showing six peaks labeled 1, 2, 3, 4, *, and 5. Right panels: SDS-PAGE analysis of the protein present in each peak in the SEC profiles, under nonreducing (without DTT) and reducing (+DTT) conditions, as indicated. Coomassie blue staining. The asterisk corresponds to a shoulder of peak 4 in the SEC profile of sE and was treated as a separate fraction in the SDS-PAGE. Molecular masses of marker proteins are listed in kilodaltons. The first two peaks of both SECs correspond to aggregates (peaks A and 1) and an aberrant disulfide-linked dimer (peaks B and 2). The remaining peaks show more heterogeneity between the two productions. In particular, peak 3 of sE expressed without prM (panel B) corresponds to a nonfunctional monomeric form that was not able to insert into liposomes in a flotation assay. Only sE derived from peak C from pr/sE and peak 5 from sE expressed without prM were used for further studies. (**C**) Isothermal titration calorimetry. sE:pr binding isotherms (bottom panel), recorded at pH 6 and 8, shown as triangles and circles, respectively, resulting from integration of the specific heats with respect to time as shown for pH 8, top panel. (**D**) Surface plasmon resonance. Example of sE:pr association and dissociation kinetics corresponding to injections of pr at 12.5 nM over immobilized sE, respectively, at pH 6 (solid line) and 8 (dashed line).

The YFV pr/sE complex was purified from the S2 supernatant as described in Materials and Methods and Fig. S1. The pr/sE complex was highly stable and only treatment at 8M urea, under nonreducing conditions, allowed separation of the two components in a gel filtration column. For comparison, we also made constructs to express sE and pr separately in S2 cells. The yields of sE produced in the absence of prM were sensibly lower and the size-exclusion chromatography (SEC) profile showed high elution heterogeneity (Fig. 2A and B), confirming the chaperone role of prM for folding of YFV sE protein. In particular, we tested peaks 3, 4, and 5 for insertion into liposomes in a flotation assay and found that only peaks 4 and 5 were functional, whereas peak 3 represents an aberrant misfolded form unable to insert into membranes. We have previously observed a similar aberrant peak for DENV E, which was not reacting with conformational EDE antibodies. We used the protein eluting in peak 5 for crystallization of the E dimer.

We used purified YFV pr and sE to measure the affinity of their interaction at pH 6 and 8 by two different methods, isothermal titration calorimetry (ITC, Fig. 2C) (24) and surface plasmon resonance (SPR, Fig. 2D). The dissociation constants (*K*_D_) obtained followed a similar trend: a *K*_D_ under 10 nM at pH 6 (8.5 nM by ITC and 6.2 nM by SPR) and about five times higher at pH 8 (58.8 nM by ITC and 19.7 nM by SPR) (Table 1), which indicated a pH-sensitive interaction that remains strong at neutral pH. In previous studies of the interaction between DENV2 pr and sE, although a *K*_D_ had not been reported, SPR experiments revealed undetectable or no binding at pH 8 (17), whereas in the case of YFV, we found a *K*_D_ value under 100 nM in these pH conditions, revealing a clearly different behavior with pH between the two viruses.

**TABLE 1:** pr–sE binding parameters^*a*^

Technique	Parameter	pH 6	pH 8
ITC	*K*_D_ (nM)	8.5 ± 3.4	58.8 ± 10.8
	ΔG (kcal.mol^−1^)	−11.0 ± 0.4	−9.9 ± 0.2
	ΔH (kcal.mol^−1^)	−16.6 ± 0.2	−16.3 ± 0.2
	TΔS (kcal.mol^−1^)	−5.6 ± 0.3	−9.4 ± 0.2
	N	0.9 ± 0.1	0.9 ± 0.1
	C value	1324	124
SPR	*K*_D_ (nM)	6.2 ± 1.8	19.7 ± 5.1
	ΔG (kcal.mol^−1^)	−11.2 ± 0.3	−10.5 ± 0.3
	*K*_on_ (10^5^ M^−1^.s^−1^)	3.8 ± 1.7	0.7 ± 0.3
	*K*_off_ (10^−4^ s^−1^)	23.6 ± 4.1	13.4 ± 0.5

^*a*^ The fit of the ITC raw data yields the change in enthalpy upon binding and the dissociation constant. G is calculated as RTln(*K*_D_), -TS is calculated as G minus H. N is the stoichiometry of the reaction and C is the ratio of the ligand concentration and the dissociation constant (24). Errors are fitting errors given by the Microcal Origin software (Microcal software, Northampton, MA, USA) for ITC and by the Biacore T200 software.

### YFV pr does not stabilize the sE dimer at low pH

**Fig 3:**
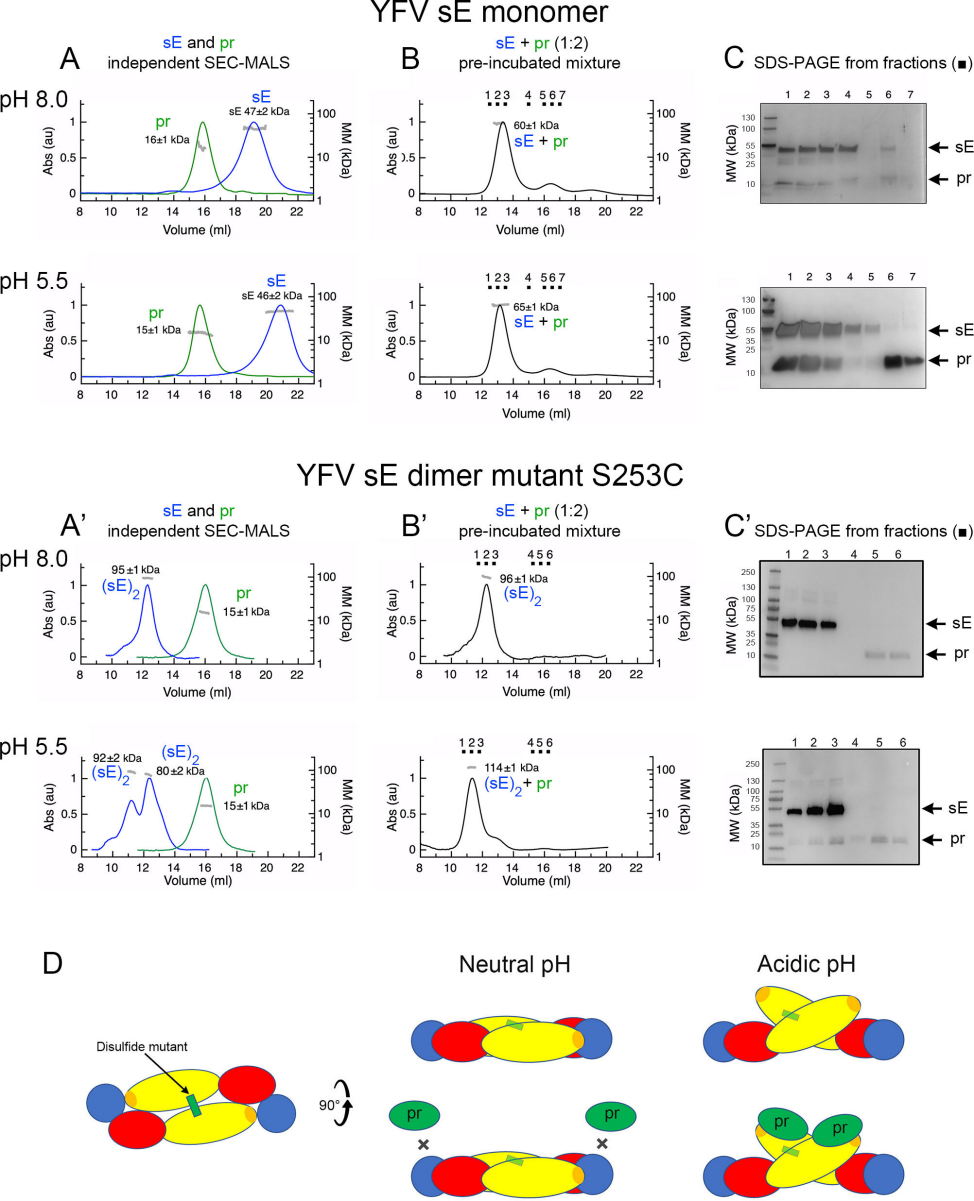
YFV pr protein binds to the sE protein monomer at both neutral and acidic pH while binding to the sE protein dimer is impaired at neutral pH. (**A, B, A', B'**) SEC-MALS elution volume profiles. Left *y*-axis: the ultraviolet absorbance normalized by setting the highest peak to 1. Right *y*-axis: molecular mass (kDa) determined by MALS, with the values for each species indicated on the corresponding peak. For both YFV sE monomer (sE) and dimer (sE)_2_: (**A–A'**) Equilibrated SEC-MALS elution profiles of isolated sE (in blue curves) and isolated pr (in green curves) equilibrated at pH 8.0 (top panel) and 5.5 (bottom panel). Note the aberrant elution volume of sE alone, which is attributed to an interaction of the fusion loop with the resin. (**B–B'**) SEC-MALS elution profiles of a mixture of sE with pr in excess (1:2 sE:pr molar ratio) at pH 8.0 (top panel) and 5.5 (bottom panel). Note that despite the molar ratio of pr:sE of 2:1, the molar extinction of pr is very weak, resulting in a small peak. The fractions analyzed by SDS-PAGE in (**C**) are indicated. (**C**) SDS-PAGE under reducing conditions and silver nitrate staining (YFV sE monomer) or (**C'**) western blot (YFV (sE)_2_ dimer) of the SEC fractions indicated in (**B–B'**) at the corresponding pH. The western blot was probed with an anti-Strep antibody of SEC fractions from the sE dimer in complex with pr at pH 8.0 and 5.5, as indicated and as described in Materials and Methods. (**D**) Schematic representation of the single cysteine mutant S253C (disulfide bond schematized as a green rectangle) and its interactions with pr at both neutral and acidic pH.

The head-to-tail E dimer observed in mature virions is recapitulated by the soluble, recombinant sE ectodomain, although it is a less stable dimer. Yet the X-ray structures show a similar head-to-tail sE dimer for most of the flaviviruses studied, since the crystals are grown at very high concentration of sE, which favors dimer formation. The most stable is the TBEV sE dimer, which, as we have shown previously, dissociates at acidic pH (21, 25, 26). This behavior is in line with that of E dimers on virions, which dissociate to expose the fusion loop at acidic pH. In a recent study, we showed that recombinant TBEV sE remained dimeric at acidic pH in the presence of pr, also recapitulating the role of pr after furin cleavage in the TGN, by maintaining the herringbone pattern of E diners at the particle’s surface despite low pH. Because pr stabilizes the sE dimer at acidic pH, it allowed the structure determination of the TBEV (pr/sE)_2_ dimer (21). To investigate the oligomeric state of YFV sE in the presence or absence of pr at different pH values, we used size-exclusion chromatography (SEC) combined with multi-angle light scattering (MALS). We observed that both, YFV pr and sE, sampled individually, eluted as monomers at pH 8 and 5.5 (Fig. 3A, top and bottom panels). Although sE has a higher molecular mass than pr, it elutes as a later peak from the SEC column, corresponding to the elution volume of a small molecule. This behavior has been previously described for DENV sE, which is a weak dimer that dissociates under the SEC column at neutral pH (22), as well as for other class II proteins in their monomeric form, and has been attributed to interactions of the exposed fusion loop with the resin (27). We reported the same behavior for TBEV sE at acidic pH (21), as the sE dimer dissociates under these conditions. The YFV pr/sE complex eluted from the SEC column at a volume corresponding to 65–60 kDa (Fig. 3B, top and bottom panels), containing both sE and pr at both neutral and acidic pH, as shown by SDS-PAGE analysis of the peak fractions (Fig. 3C, top and bottom panels). For TBEV, pr binds to the sE dimer only at acidic pH (21). To gauge the general behavior of pr in complex with sE in other flaviviruses, we carried out parallel tests with pr and sE from DENV2 and ZIKV (Fig. S2). These analyses revealed that pr does not stabilize an sE dimer for neither of these two viruses at acidic pH, and only monomeric pr/sE forms were detected, in line with what we observed for YFV sE. The main difference is that pr does not show detectable binding to sE at pH 8 for ZIKV or DENV2, contrary to YFV (Table 2).

**TABLE 2:** sE and pr oligomerization states and pr–sE binding at pH 5.5 and 8.0^*a*^

sE variant	pH 5.5	pH 5.5 + pr		pH 8.0	pH 8.0 + pr	
	sE oligomer MM (kDa)	sE oligomer MM (kDa)	sE-pr binding	sE oligomer MM (kDa)	sE oligomer MM (kDa)	sE-pr binding
YFV	Monomer 46 ± 2	Monomer 65 ± 1	Yes	Monomer 47 ± 2	Monomer 60 ± 1	Yes
YFVd	Dimer 92 ± 2	Dimer 114 ± 1	Yes	Dimer 95 ± 1	Dimer 96 ± 1	No
ZIKV	*Aggregation*	Monomer 54 ± 3	Yes	Dimer 91 ± 2	Dimer 91 ± 1	No
DENV2	Monomer 45 ± 1	Monomer 48 ± 1	Yes	Monomer 51 ± 4	Monomer 49 ± 1	nd
DENV2d	Dimer 92 ± 3	Dimer 135 ± 7	Yes	Dimer 91 ± 4	Dimer 91 ± 2	No
TBEV	Monomer 52 ± 1	Dimer 93 ± 1	Yes	Dimer 95 ± 2	Dimer nd	No

^*a*^ nd, not determined; YFVd, YFV sE S253C mutant; DENV2d, DENV2 sE A259C mutant. See corresponding Fig. 3 for YFV and YFVd and Fig. S2 for ZIKV, DENV2, and DENV2d. Values for tick-borne encephalitis virus (TBEV) are extracted from reference (21).

**Fig 4:**
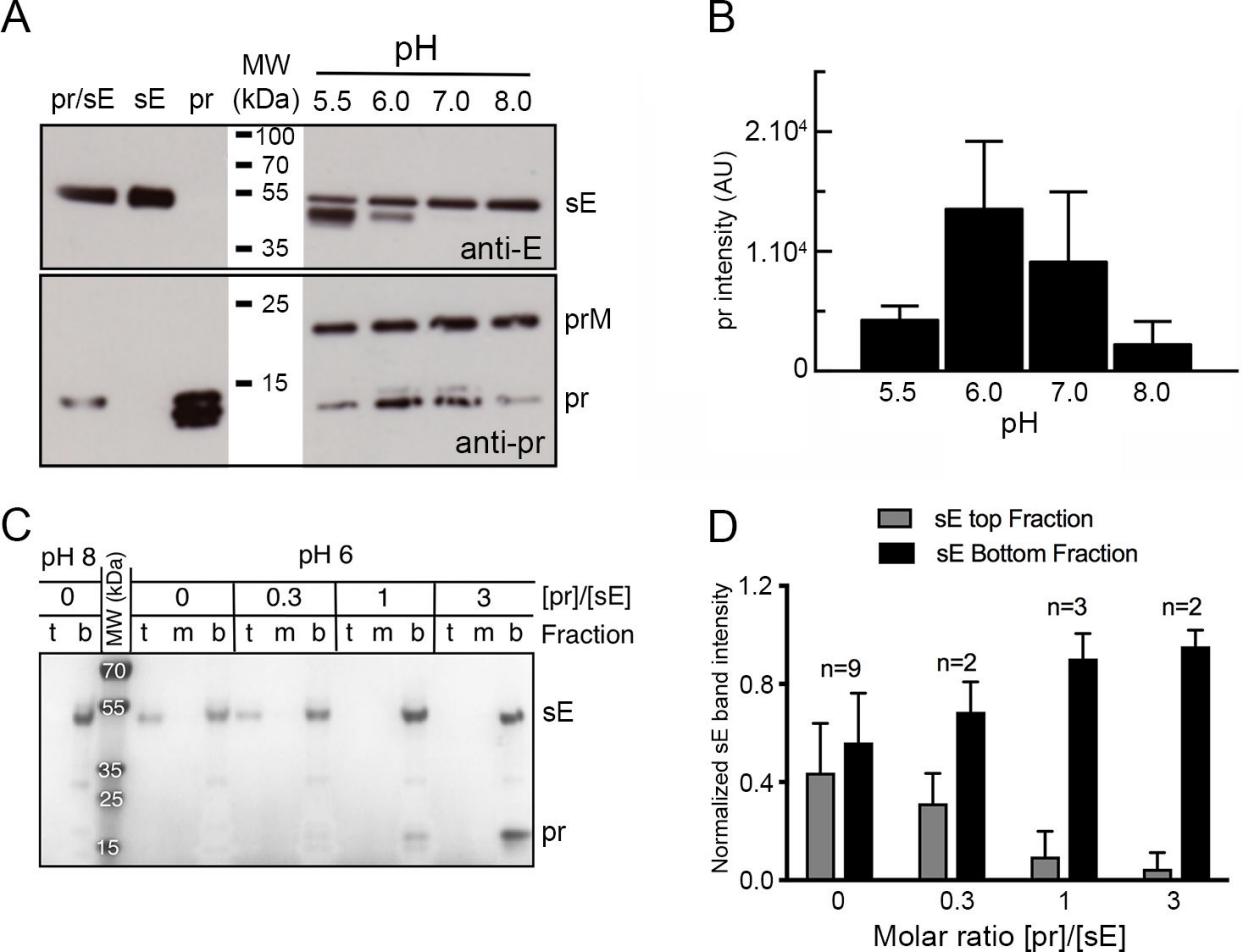
Binding of YFV pr to the virion is enhanced at low pH and prevents sE insertion into membranes at 1:1 stoichiometry. (**A**) Binding of exogenous pr to YFV 17D viral particle. Western blot with E- or pr-specific antibodies. About 10^8^ ffu of virus was mixed with an excess (1:50) of exogenous purified pr protein and incubated in buffer at different pHs. The complex was then pelleted by ultracentrifugation and analyzed by SDS-PAGE and western blot. The pr doublet is probably the result of the heterogeneous processing of this glycosylated protein. Note that the prM band corresponds to immature virus present in the viral preparation. (**B**) Histogram representing the values of pr band intensity from two experiments. (**C**) Co-floatation assay. Five mg of purified sE protein was mixed with different amounts of purified pr protein and with liposomes (refer to Materials and Methods for lipid composition). After addition of buffer at the indicated pH and overnight incubation at 30°C, the protein–liposomes mixture was separated on an Optiprep gradient. Coomassie-stained SDS-PAGE of top (**t**), medium (**m**), and bottom (**b**) fractions is shown. sE protein liposome co-flotation was performed at pH 8 (left columns) and at pH 6 in the presence of pr at sE:pr molar ratios 0.3, 1, and 3 (right columns). (**D**) Histogram of normalized sE band intensity from top and bottom fractions to the amount of sE present in the bottom fraction at pH 8. Several flotation assays were included in the calculation using ImageJ software. Errors are standard deviations calculated from at least two experiments.

As mentioned earlier, at the surface of mature flavivirus particles, E is always dimeric at neutral pH. We found that YFV sE makes a similar head-to-tail dimer only under crystallization conditions (which require a very high protein concentration), as observed for sE of DENV and JEV (22, 28, 29). In the case of DENV, we had previously identified a cysteine mutation (at position 259 in sE from DENV1, 2, and 4, and position 257 in DENV3) that locks the protein as a head-to-tail dimer as on mature virions (30). We therefore engineered the corresponding cysteine mutation (S253C in YFV sE) to induce formation of an inter-protomer disulfide bond across the two sE protomers (Fig. S3). Investigation of the interactions of YFV pr with this mutant showed binding only at acidic pH (Fig. 3A through C, bottom panels) and not at pH 8 (Fig. 3A through C, top panels). The gels shown in panel 3C’ were run under reducing conditions. We conclude that the head-to-tail dimer does not resist low pH treatment and exposes the tip of domain II to allow for pr binding, despite the covalent linkage of the two protomers, as illustrated diagrammatically in Fig. 3D. In conclusion, based on the analysis of several mosquito-borne flaviviruses (ZIKV, DENV2, and YFV), the pr-binding site on E is not accessible on a head-to-tail dimer as on virions at neutral pH. Yet, pr must bind the E dimers on virions in the TGN to protect from premature fusion at acidic pH, as posited in the accepted model of particle maturation for all flaviviruses (31). To confirm this interpretation, we mixed pr with mature YFV 17D particles in a stoichiometric excess of 50:1 pr:E at various pH values, and measured the amount of pr brought down upon pelleting of the virion by ultracentrifugation (Fig. 4A). We used YFV 17D virus because of safety reasons since the vaccine strain can be manipulated under BSL2 conditions. This experiment showed very little pr co-precipitating with the virus particles at pH 8, and a maximum of co-precipitation at pH 6, suggesting a pH-dependent exposure of the pr-binding site on virions (Fig. 4B).

### YFV pr protein blocks sE insertion into membranes at low pH

We tested the effect of the presence of pr on the interactions of sE with membranes by measuring co-flotation with liposomes in a density gradient at neutral and acidic pH (Fig. 4C and D). At pH 8, we detected no interaction with liposomes, and sE remained at the bottom of the gradient despite the fusion loop being exposed in the monomers (Fig. 4C, left column). Instead, at pH 6, we observed about 45% of sE floating at the top fractions together with the liposomes (Fig. 4C and D). In the presence of pr, we found a dose-dependent inhibition of sE/liposome co-flotation, such that at a molar ratio of 1:1, there was no sE protein in the top fractions, in line with the *K*_D_ of 10 nM or less of the pr/sE complex at pH 6 (Table 1). These data show a different behavior compared to previous results with DENV2, where a 10-fold molar excess of pr was required to inhibit liposome insertion in a similar assay (16), again indicating that the interaction of pr with the E protein is stronger in the case of YFV.

### pr blocks the fusion of YFV particles with liposomes at low pH

**Fig 5:**
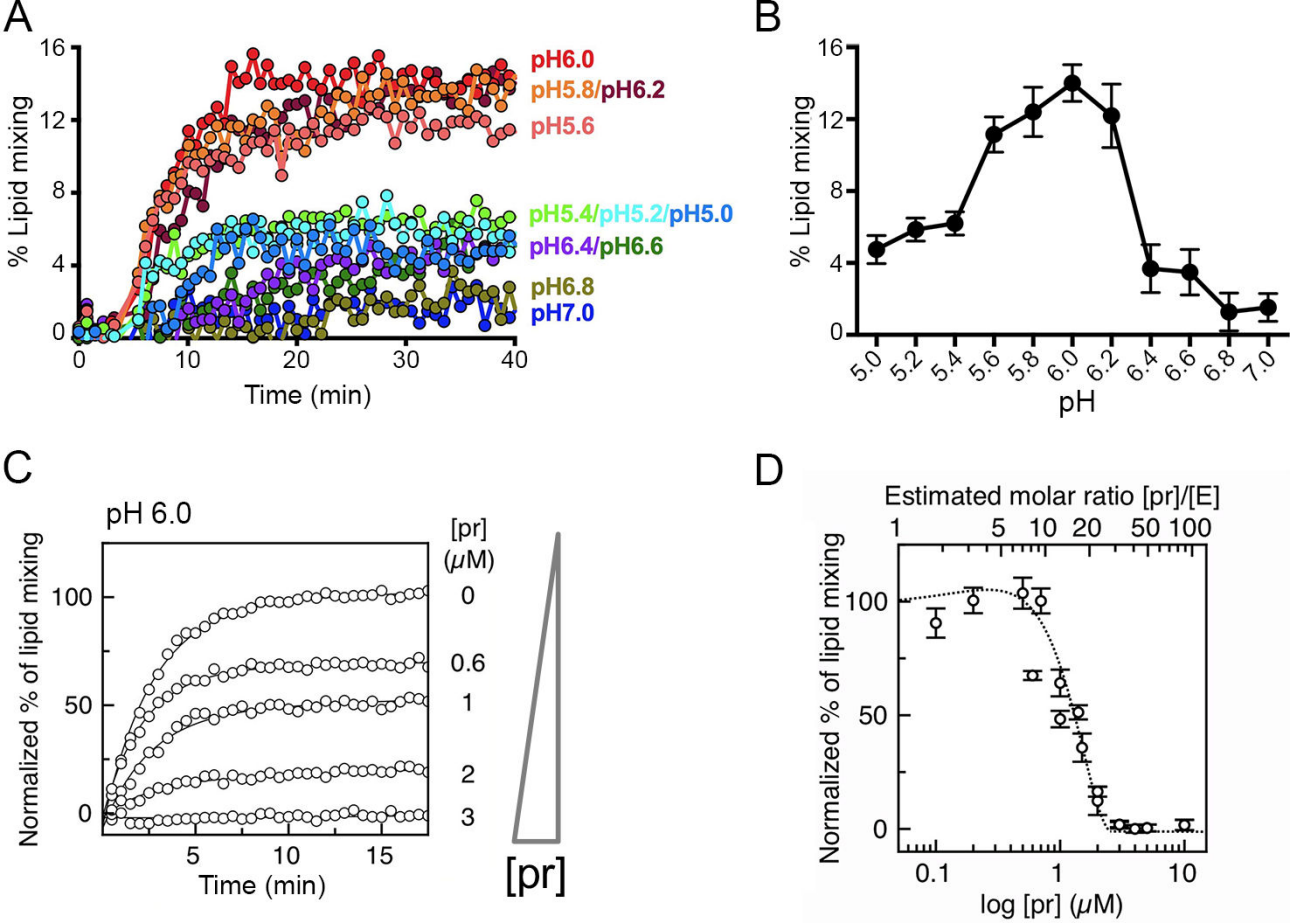
A 10-fold molar excess of pr over E is required to block YFV viral fusion. (**A**) Lipid mixing assays between YFV 17D virus and NDB/Rho-labeled liposomes recorded from pH 5.0 to pH 7.0 at every 0.2 pH units. About 10^7^–10^8^ ffu of purified virus was mixed with 500 nM labeled liposomes resuspended in buffer at different pH. Fluorescence emission was recorded for 40 min in a multiplate reader fluorimeter (Tecan M1000) and the reaction was stopped by the addition of detergent to measure the maximal (100%) signal. The represented extent of lipid mixing was related, for each pH, to the maximum signal recorded upon lipid dilution by detergent addition (see Materials and Methods). (**B**) Plot of mean fluorescence signal registered at min. 7–37 for each pH. (**C**) Representative curves of normalized NBD fluorescence intensity recorded at 535 nm as a function of pr concentration, lines are monoexponential fits to the data. Sample without pr was considered 100%. About 10^7^–10^8^ ffu of purified virus was mixed with an increasing amount of purified pr and incubated at 37°C for 30 min. Virus/pr mixture was then added to 200 nM liposomes in MES buffer pH 5.5. The final pH of the mixture was pH 6. Fluorescence emission was recorded for 30 min in a plate reader fluorimeter (Tecan M1000) and the reaction was stopped by the addition of detergent to measure the maximal signal. (**D**) Percentage of lipid mixing as a function of pr concentration. Top *x*-axis corresponds to the [pr]/[E] molar ratio as estimated by western blot (see Materials and Methods and Fig. S4). The dashed line is shown as a guide.

The fusion of enveloped virus particles with artificial membranes can be measured using lipid mixing assays. We measured the fusion of virus particles to liposomes by fluorescent energy transfer (see Materials and Methods for details of the assay). The fluorescence profile observed upon mixing YFV 17D virus with the NBD/rhodamine-labeled lipids at different pH values is displayed in Fig. 5A. Fluorescence dequenching is optimal between pH 5.6 and 6.2 and is negligible at neutral pH. A plot of the mean intensities reached at each pH value shows a peak of lipid mixing at around pH 6 (Fig. 5B). We therefore used pH 6 to test the inhibition of lipid mixing by recombinant pr added at different pr:E stoichiometries to the virus preparation before mixing with liposomes and lowering the pH. These experiments showed a dose-dependent inhibition of the reaction by exogenous pr (Fig. 5C). We quantified the relative stoichiometry of pr:E by western blot as described in the Materials and Methods section (see Fig. S4). Different from the results with purified YFV sE insertion into liposomes (Fig. 4C), we observed a requirement of at least 10-fold excess pr to obtain 100% inhibition of lipid mixing (Fig. 5D). Because in these experiments the pH is lowered in the presence of liposomes, we interpret this difference as a kinetic effect, in which only the presence of a high excess of pr leads to sufficient binding of the E protein before the dimers have dissociated and the fusion loop has inserted into the liposomes.

### Conformation of the YFV head-to-tail sE dimer

**Fig 6:**
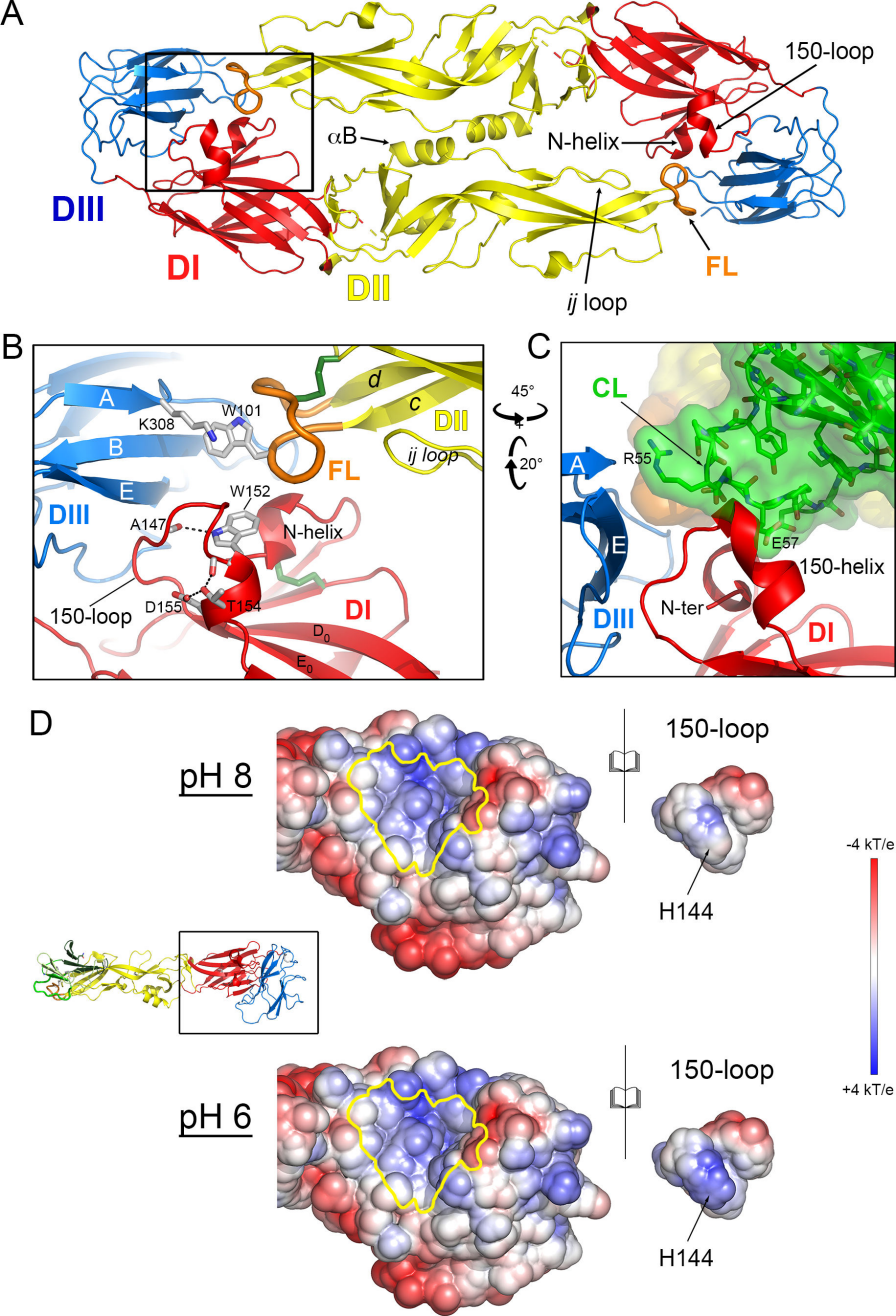
The 150-loop in E dimer interferes with pr binding at neutral pH. (A) Ribbon view of the crystallographic YFV sE dimer colored according to the definition described in Fig. 1. The fusion loop in orange is buried at the dimer interface against 150-loop and *ij* loop, as indicated. (**B**) Close view showing the proximity of the 150-loop with the N-terminus of E (N-helix). (**C**) Close view of modeled binding of pr protein on the sE dimer showing the clash of the pr capping loop (CL in green) with the 150-loop (150-helix) and the N-terminal of E. The superposition of pr/sE structure (PDB 6EPK, this work) on sE dimer was done using the tips of E-domain II containing the fusion loop (beta-strands *b,c,d,* and *i,j*; see Fig. 1). (**D**) The 150-helix packs against a positively charged internal surface of the E protein. To illustrate this interaction, we extracted the 150-loop from protein E, and calculated the surface electrostatic potential of the remainder at pH 8 (top) and 6 (bottom). The figure shows an open-book representation of the 150-loop, for which we also determined its surface electrostatic pH at both pH values. This image suggests that the protonation of H144 in the 150-helix could introduce electrostatic repulsion and result in a shift of the 150-helix, at low pH, thereby removing the clash and allowing pr to bind.

There are two main sE dimer interfaces, the first by the dimer axis and the second one involving the fusion loop, away from the dimer axis. The first interface involves an antiparallel interaction around helix αB (Fig. 6A), including inter-protomer hydrogen bonds, some of which involving main-chain/main-chain interactions. In the second interface, the fusion loop at the tip of DII packs against DI and DIII of the opposite protomer in the dimer (Fig. 6B), as described previously for other flaviviruses. The side chain of the strictly conserved Trp101 of the fusion loop is covered by that of Lys308 of DIII, while the fusion loop main chain is partially tucked in between two short helices in DI, the N-terminal helical turn (N-helix in Fig. 6B) and a helix in the 150-loop (the 150-helix) (Fig. 6B). The amino acid sequence of the 150-loop is highly variable across flaviviruses, in most of which it is N-glycosylated. In YFV, it is N-glycosylated in only a few attenuated strains (32). Residues 149–155 form the 150-helix, highly exposed at the dimer surface. The side chain of Trp152 in the 150-helix packs against the N-terminal end of the polypeptide chain, which is buried underneath (Fig. 6B). The positively charged N-terminus is neutralized by a salt bridge and hydrogen bond with the strictly conserved Asp42 side chain, which is also buried. This interaction is part of a network of hydrogen bonds also involving residues from DIII.

Superposition of DII of the YFV pr/sE complex on its counterpart on the sE dimer structure revealed a steric clash with the 150-helix, indicating that pr binding cannot take place with the 150-loop in the conformation observed in the sE dimer at neutral pH. We had previously observed the 150-loop in an alternative conformation on the TBEV sE dimer at low pH to accommodate pr (21). Thus, it seems likely that the 150-loop of YFV will also adopt an alternative conformation at low pH, thereby promoting pr binding. An alternative conformation of the 150-helix might take place upon protonation at acidic pH, as suggested by the surface electrostatic potentials displayed in Fig. 6D. The second turn of the 150-helix is constrained by a hydrogen bond between the Thr154 and Asp155 side chains (Fig. 6B). Importantly, one of the virulence determinants of YFV identified in a hamster model mapped to these two residues as replacement for Ala resulting in increased virulence in hamsters (33). Because the side chain of Trp154 is important for the packing of the 150-helix at the E protein surface, this observation suggests that altering the conformation of the helix may affect the interaction of E and pr. The correct structuring of the helix is likely to be important for a correct switch between the two conformations, at neutral and low pH, such that only in the second conformation pr can bind. In summary, the 150-helix is highly exposed and structured at the dimer surface, in a region important for stabilizing interactions between DI and DIII, and the fusion loop on the adjacent dimer subunit.

## DISCUSSION

We describe here the structure of sE from the YFV Asibi strain alone and in complex with pr in the absence of an artificial linker keeping the complex together, as in the case of DENV. We show that in the absence of pr, sE crystallizes as the canonical head-to-tail dimer observed for other flaviviruses, and that the complex with pr does not form such a dimer, in stark contrast with the TBEV counterpart. The pr/sE interaction is strong even at neutral pH, implying that a simple rise in the environmental pH to neutral values upon reaching the extracellular milieu is not sufficient for YFV particle activation for subsequent fusion with an endosomal membrane. These results are therefore in line with our previous findings on TBEV, which indicated that particle activation involves pr ejection from the dimer by a conformational change of the 150-loop in the adjacent protomer of the E dimer, which knocks pr out of its binding site on DII. We further found that the recombinant sE dimer of other *Aedes* mosquito-species-borne flaviviruses is also not stabilized at low pH by pr. For these viruses, the affinity of the sE protomers for homodimer formation is weaker than for TBEV, and the addition of pr leads to monomeric pr/sE complexes only. We hypothesize that the *Culex* mosquito-species-borne encephalitic viruses of the JEV serocomplex are likely to behave in the same way, as they were shown to also make weak dimers, only detected under crystallization conditions (28).

A comparison of the X-ray structure of the YFV pr/sE complex with its counterparts from DENV2 and TBEV does not provide an obvious explanation for the difference in affinity of pr for E DII, as we observe a similar number of interactions in both cases and a similar buried surface area. For TBEV, however, the affinity between pr and E DII has not been measured, since sE behaves as a dimer at neutral pH and pr cannot bind, while at acidic pH it binds tightly at the sE dimer interface with a very high electrostatic component (21).

Formation of the herringbone pattern by (prM/E)_2_ dimers at acidic pH has been postulated to be a general property of flaviviruses. Dimer stability on virions therefore involves regions outside the sE moiety, most probably stabilized at neutral pH by interaction with the E stem region (absent in sE) and the M ectodomain, and probably also by lateral interactions between E dimers at the particle surface. The structure of the E dimers appears to be pH-modulated by the 150-loop, which is an insertion in comparison to the proteins from other class II viruses, such as alphaviruses and bunyaviruses (34). This insertion may have been evolved to accommodate the use of prM, which was derived from a cellular DnaJ chaperone protein to function in membrane fusion control and maturation (21).

The structural studies on TBEV showed that the 150-loop changes conformation at acidic pH to allow for pr binding at the sE dimer interface. We observed a clash of the 150-loop in the YFV sE dimer in the neutral pH conformation, which would block binding pr at neutral pH. In agreement with this observation, we showed that there is no significant pulldown of pr by intact virions at neutral pH, whereas it is significant upon exposure at low pH. We interpret this finding as pr binding to virions on the herringbone pattern of dimers, on which the 150-loop adopts a conformation compatible with pr binding, as it had been shown for TBEV.

In conclusion, we describe molecular interactions regulating a crucial process in flavivirus maturation. While the basic organization of the interactions is common to all flaviviruses, each flavivirus species appears to modulate them differently. We thus found for YFV a stable association with pr also at neutral pH, showing that its release from the mature particle is not via a passive pH-dependent loss of affinity as posited in the DENV paradigm, but that it is expelled in a local rearrangement of the E dimers. Furthermore, as shown earlier (28), our data indicate that the head-to-tail E dimers have different requirements for stability depending on the species, and the ectodomain appears sufficient only in TBEV. Additional changes in the peri-membranous region of the E and M proteins are likely to also play a role in particle activation, allowing alternative conformations of the E 150-loop still in the context of a head-to-tail dimer. The contributions of these other regions remain open to further investigation.

## MATERIALS AND METHODS

### Recombinant pr/sE protein production

The YFV Asibi pr/sE and sE constructs were cloned onto a pMT-derived vector (35). This vector allows expression of the gene of interest downstream the *Drosophila* BiP signal peptide and upstream a StrepTag, used for purification purposes. The details of the purification of the different proteins are in the Supplemental Material.

### Crystallization, data collection, refinement, and model building

A detailed description of the structural data is in the Supplemental Material.

The final models of pr/sE and sE dimer contain all amino acids of YFV sE (1-392) and residues 1 to 80 of pr. Data collection and refinement statistics as well as the MolProbity (36) validation statistics for all the two structures are presented in Table S1.

### Structural analysis

The polar contacts in Table S2, the accessible surface area (ASA) and the buried surface area (BSA) in Table S3, were computed with the “Protein interfaces, surfaces, and assemblies” server PDBePISA (37) (http://www.ebi.ac.uk/pdbe/prot_int/pistart.html). The multiple sequence alignments in Fig. 1 were calculated using DALI (http://ekhidna2.biocenter.helsinki.fi/dali) (38) and displayed with ESPript (http://espript.ibcp.fr). The figures of the structures were prepared using the PyMOL molecular graphics system (Schrodinger) (pymol.sourceforge.net). Electrostatic surfaces were computed using APBS, PDB2PQR, and PROPKA softwares via the server (39) (https://server.poissonboltzmann.org).

### Multi-angle static light scattering-SEC

MALS studies were performed using a SEC Superdex 200 column (GE Healthcare) previously equilibrated with the corresponding buffer (see Supplementary Material for a detailed description).

### Liposome assays

Liposomes used for lipid mixing and co-flotation assays were prepared by following a modified film-hydration protocol (40). See Supplemental Material for detailed protocols.

### ITC and SPR

The protein samples were extensively dialyzed prior to ITC. The measurements were performed in duplicate, using 50 mM Tris, 50 mM MES (pH 6, 7, and 8), and 150 mM NaCl at 25°C (41) (Table 1).

The affinity of the sE protein for the pr peptide was measured by SPR using a Biacore T200 system (GE Healthcare Life Sciences) equilibrated at 25°C.

See Supplemental Material for a detailed description of the experiments.

### Virus stock generation, purification, and titration

Yellow fever 17D (YFV 17D) viral stocks were derived from pACNR/FLYF plasmid (42) containing the full-length infectious YF17D-204 genome. See Supplemental Material for a detailed description.

### Co-precipitation of purified pr with YFV 17D virus

Cell culture supernatant containing 10^8^ total particles of YFV 17D virus was pelleted over a 20% sucrose cushion and resuspended in 100 µL of TNE buffer (10 mM Tris pH 8, 150 mM NaCl, 1 mM EDTA). An excess of purified pr peptide was added to the virus (ratio 1:50) and the pH was changed with phosphate/citrate buffer to pH 5.5–6–7–8. After 30 min incubation at 37°C the complex was pelleted in a SW55 rotor at 100 kg for 1 h and loaded on a 12% SDS gel. Antibody E21.3 was used in western blot to detect the viral E protein and antibody A3.2 was used to detect the pr protein. Band intensities were calculated in ImageJ software (43).

## Data Availability

Coordinates and structure factor amplitudes for YFV strain Asibi pr/sE and sE dimer have been deposited in the Protein Data Bank, respectively, under the accession numbers 6EPK and 8OFN. The accession numbers are as follows: tick-borne encephalitis virus (TBEV, strain Neudoerfl), GenBank accession number U27495; dengue virus type (DENV2, strain 16681), GenBank accession number M19197; and yellow fever virus (YFV, strain Asibi), GenBank accession number AY640589.

## SUPPLEMENTAL MATERIAL

Supplemental material mbio.00706-23-s0001.pdf Additional Material and Methods, Figures S1 to S4, and Tables S1 to S3.

ASM does not own the copyrights to Supplemental Material that may be linked to, or accessed through, an article. The authors have granted ASM a non-exclusive, world-wide license to publish the Supplemental Material files. Please contact the corresponding author directly for reuse.
