# Supplementary material for: New insight into flavivirus maturation from structure/function studies of the yellow fever virus envelope protein complex
Source: mBio. 2023 Aug 21;14(5):e00706-23. doi: 10.1128/mbio.00706-23 (PMC10653854; doi:10.1128/mbio.00706-23)
Supplement: Supplemental material — Additional Material and Methods, Figures S1 to S4, and Tables S1 to S3. [file mbio.00706-23-s0001.pdf]

SUPPLEMENTAL MATERIAL AND METHODS

Recombinant pr/sE protein production. The sequence encoding for prM and the ectodomain of E for the Asibi strain (NCBI AY640589) was taken from pACNR-113.16 (Rice and Barba-Spaeth, unpublished). The single cysteine mutation S253C was introduced to generate a disulfide stabilized dimeric protein. All the constructs had a stop codon after position 392 of E (i.e., immediately after DIII). *D. melanogaster* S2 pseudo-clonal pools were generated by co-transfection with a pCoPURO (1) by Effectene transfection (QIAGEN). For expression, cells were induced at a density of $6x10^6$ cells per mL with 500 μM Cu_2SO_4 for 10 days or 5 μM $CdCl_2$ for 7 days. The supernatant was then harvested, concentrated on a Vivaflow 200 concentration system with a 10 kDa-cutoff membrane (Sartorius). The pH of the concentrated supernatant was adjusted to 8 with 100 mM Tris-HCl. Avidin was added to a final concentration of 1 μg/mL. The produced protein was then captured on a StrepTactin column, washed, and eluted with binding buffer (100 mM Tris-HCl pH 8, 150 mM NaCl, 1 mM EDTA) supplemented with 2.5 mM desthiobiothin. The peak obtained by affinity chromatography was further purified by size-exclusion chromatography (SEC) using a Superdex 200 16/60 column (GE Healthcare) with 20 mM Tris-HCl pH 8 and 150 mM NaCl. The purified protein was then dialyzed against 10 mM Tris-HCl pH 8 and loaded on MonoQ 5/15 column (GE Healthcare) to be eluted using two steps, one at 240 mM followed by one at 400 mM NaCl in the same buffer.

The purified YFV pr/sE complex was highly stable, resisting separation by anion exchange chromatography in a NaCl gradient (see Fig. S1). Yet this procedure led to elution of the sample in two separate peaks, one containing the pr/sE complex and the other only sE (termed sE') (Fig. S1). Functional analysis of sE', which eluted at 400 mM NaCl, revealed it to be a non-functional from that was unable to insert into liposomes at acidic pH (see experimental details below). The protein eluting in the peak at 240 mM NaCl was further used to separate sE and pr.

To dissociate the pr/sE complex under non-reducing conditions, we used denaturing conditions, by adding 8 M urea to the solution. The dissociated pr and sE proteins (10 kDa and 47 kDa, respectively) were separated by SEC in 10 mM Tris-HCl pH 8, 6 M urea and 1 M KSCN. The samples were collected and dialyzed overnight against 20 mM Tris-HCl pH 8 to remove all traces of urea. A final SEC purification on a Superdex 200 16/60 in 20 mM Tris-HCl pH 8 and 150 mM NaCl was done to obtain pure, refolded pr and sE.

For comparative studies, ZIKV sE (strain PF13), DENV2 sE (SG strain) and the DENV2 sE A259C mutant (16681 strain, equivalent to the YFV S253C SE mutant), were produced as described earlier. The SEC profile of the S253C mutant showed the presence of high-molecular weight aggregates and peaks corresponding to monomeric protein, but a fraction of the protein was produced as a disulfide linked dimer as shown by MALS and SDS-PAGE analysis (Fig. S3). The affinity chromatography step was followed by size exclusion chromatography using Superdex 200 10/300 GL column equilibrated in 50 mM Tris-HCl (pH 8) and 150 mM NaCl. In parallel, pr from YFV, ZIKV (PF13 strain) and DENV2 (16681 strain) was expressed in the same way and purified with a streptactin column followed by a single SEC step using Superdex 75 10/300 GL column equilibrated in 50 mM Tris-HCl (pH 8) and 300 mM NaCl.

Crystallization.

Crystallization of the pr/sE complex. Initial screening of crystallization conditions was carried out by the vapor diffusion method using a MosquitoTM nanoliter-dispensing system (*TTP Labtech, Melbourn, United Kingdom*) following established protocols (2). After optimization, we obtained crystals diffracting up to 3Å resolution but an analysis of the intensity distribution of the diffraction data revealed merohedral twinning of the crystals, with an apparent space group $P4_122$. To overcome this problem, we introduced an additional purification step using denaturation / renaturation of the heterodimer under non-reducing conditions. The reassembled pr/sE complex, concentrated to 3 mg/mL in 20 mM Tris-HCl pH

8 and 150 mM NaCl, crystallized in 100 mM Tris-HCl pH 8 in a range of 1.2-1.8 M Li_2SO_4. For cryoprotection, the crystals were soaked in mother liquor plus 25% glycerol and flash-frozen under liquid nitrogen.

Crystallization of YFV sE. The purified protein in 20 mM Tris-HCl pH 8, 150 mM NaCl was adjusted to a concentration of 3.2 mg/mL. Crystals grew in 1.26 M $(NH4)_2SO_4$ and 0.1 M HEPES pH 7.5. These crystals belong to tetragonal space group $P4_32_12$ and had an anisotropic diffraction pattern with a resolution extended to 3.5Å in the best direction.

Structural Data (collection, refinement, model building and analysis). The structure of the YFV pr/sE complex was determined by molecular replacement with program AMoRe (3) using the atomic models of TBEV sE (PDB 1SVB, 43.4% sequence identity) (4) and DENV2 pr (PDB 3C5X, 34.6 % sequence identity) (5). The model was then manually adjusted into the electron density with COOT (6) and refined with BUSTER-TNT (7, 8) and PHENIX.REFINE (9). 2-fold non-crystallographic symmetry, target restraints (10) and TLS-based refinement (11) were applied.

The diffraction data from the sE crystals were elliptically truncated and corrected for anisotropic diffraction using using the STARANISO server (12). The unmerged protocol resulted in data reaching 3.48Å along the best axis and 4.87Å along the worst direction, with a cutoff threshold of 1.5 of the local I/σ(I) ratio. The model for sE derived from the pr/sE structure was used for molecular replacement to determine the structure. The final structure showed two independent molecules in the crystal asymmetric unit, generating the classical head-to tail sE dimer through a 2-fold crystallographic symmetry axis. Refinement was done as outlined above for the pr/sE complex.

The final models of pr/sE and sE dimer contain all amino acids of YFV sE (1-392) and residues 1 to 80 of pr. Data collection and refinement statistics as well as the MolProbity (13) validation statistics for all the two structures are presented in Table S1.

Multi-angle static light scattering-Size exclusion chromatography. SEC runs were performed at 25 °C with a flow rate of 0.4 mL/min, protein injection concentration was 100 µg. Online MALS detection was performed with a DAWN-HELEOS II detector (Wyatt Technology, Santa Barbara, CA, USA) using a laser emitting at 690 nm. Online differential refractive index measurement was performed with an Optilab T-rEX detector (Wyatt Technology). Data were analyzed, and weight-averaged molecular masses (Mw) and mass distributions (polydispersity) for each sample were calculated using the ASTRA software (Wyatt Technology). For each virus, equilibration buffers for addressing the effect of pH for sE, pr and the sE:pr complex were the three-component buffers, 100 mM Tris-HCl, 50 mM MES, 50 mM sodium acetate and 150 mM NaCl, at pH 5.5 or pH 8. The sE:pr complex, in 1:2 molar ratio (monomer:monomer molar ratio), were prepared by incubation in the corresponding three-component buffers. Buffer exchange was performed by extensive dialysis of the sample, 12 h stirring at 4 °C and two 500 mL buffer replacement in 10 kDa molecular weight cut-off dialysis membranes (Spectrum). SEC fractions of sE:pr complexes at pH 5.5 or 8 were further analyzed by Coomassie blue or silver nitrate SDS-PAGE or by western blot using an anti-strep antibody for simultaneously detection of both E and pr proteins.

Liposomes preparation. Liposomes were prepared by freeze-thaw and extrusion through 100nm pore size polycarbonate filters (Whatman 800309). Briefly, chloroform solutions of DOPC, DOPE, SM, Cholesterol, NBD-PE and Rho-PE, were pooled using glass graduated syringes (Hamilton) in borosilicate tubes at a molar ratio of 1:1:1:3:0.1:0.1, respectively, and a total lipid concentration of 10 mM. The fluorescent lipids (NBD-PE and Rho-PE) were omitted in the preparation of liposomes for co-floatation assays. The organic solvent was evaporated in the tube under a steam of N2 gas yielding a thin lipid film which was further dried by Speed-Vac (Thermo Electron, RVT400), 1 hour at room temperature. The lipid film was resuspended in 20 mM HEPES pH 7, 50 mM NaCl degassed buffer, by vortexing in presence of 180 µm acid washed glass beads (Sigma). The resulting opaque solution, composed by multilamellar vesicles, was subjected to 10 cycles of liquid N2 flash freeze-thaw

and extruded using a polycarbonate filter of 100 nm pore size until translucency, more than 20 extrusion cycles. The hydrodynamic diameter and homogeneity of the sample was controlled by dynamic light scattering. The final lipid concentration was determined by a using NBD-PE absorbance at 460 nM and a standard curve. The liposomes were stored under N2 (gas) for up to three weeks at 4ºC. All the lipids as well as the extrusion system were purchased from AVANTI Polar Lipids (USA). Abbreviations: DOPC: 1,2-dioleoyl-sn-glycero-3-phosphocholine; DOPE: 1,2-dioleoyl-sn-glycero-3-phosphoethanolamine; SM: Sphingomyelin (brain, porcine); NBD-PE: 2-dioleoyl-sn-glycero-3-phosphoethanolamine-N-(7-nitro-2-1,3-benzoxadiazol-4-yl); Rho-PE: 1,2-dioleoyl-sn-glycero-3-phosphoethanolamine-N-(lissamine rhodamine B sulfonyl) (ammonium salt).

sE-liposomes co-floatation assay. Renatured sE and pr proteins were mixed at different molar ratio and incubated for 10 min at RT before addition of liposomes. The mixture was further incubated for 10 min at RT before overnight incubation at 30°C under acidic conditions. The liposomes were then separated by ultracentrifugation on an Optiprep (Proteogenix 1114542) continuous 0-30% gradient. Aliquots from top and bottom fractions were analyzed by Coomassie gel or by western blot gels using in house produced anti-YFV E (E21.3) mouse monoclonal antibody. At least two and up to nine experiments were performed for the different molar ratios tested, the bands intensity from top and bottom fractions were analyzed by ImageJ software and plotted as ratio to total protein present in each floatation assay.

pH triggered lipid mixing, pH and pr titrations. For our lipid mixing assay, we used a system based on fluorescence resonance energy transfer between the fluorophores 7-nitro-2-1,3-benzoxadiazol-4-yl (NBD) and rhodamine covalently coupled to lipids (Duzgunes, Struck). The fluorescence is quenched at high concentration of the two fluorophores in the liposomes and becomes de-quenched upon dilution into the lipids derived from the viral membrane after fusion, allowing to follow the lipid merger reaction. Mixture reaction for pH titrations: 10 µl of purified virus (10^9-10^{10} ffu/mL) were added to 100 µl of 500 nM labelled

liposomes diluted into 300 mM citrate-phosphate buffer at pH 5.0, 5.2, 5.4, 5.6, 5.8, 6.0, 6.2, 6.4, 6.6, 6.8 or 7.0. Mixture reaction for pr titrations: 10 µL of purified virus (10^9-10^{10} ffu/mL) were incubated in a multi-well plate (Greiner) with increasing amounts of purified pr protein for 30 min at 37ºC in 100 mM Tris-HCl pH 7.5 and 150 mM NaCl, 50 µL total volume. Subsequently, 100 µl of 200 nM NBD-PE and Rho-PE labelled liposomes in 50 mM MES pH 5.5, was added to the virus/pr complex using a multichannel pipette and gently mixed three times prior data collection (average dead time 40s). The pH after the mixture was 6.0±0.2. For both titration assays, the emission fluorescence of NBD was recorded in a multi-plate reader fluorimeter (Tecan M1000), with an excitation and emission wavelength of 460 nm and 539 nm and slits widths of 10 nm and 20 nm, respectively, during more than 3 times the end of the lipid mixing reaction (~10 minutes) at 25ºC. The maximum NBD emission signal was recorded by addition of 10 µl of 2.5% C13E8 (Polyoxyethylene(8)tridecyl Ether, Anatrace) for 10 minutes. For pr titrations, a mock reaction (no virus) was performed for each pr concentration by using the same virus buffer and used as reference signal. The extent of lipid mixing was calculated from the recorded intensities (I) by (I-I0)/(I100-I0), with I0 the initial intensity and (I100) the maximum NBD emission signal recorded upon addition of detergent. The % of lipid mixing was calculated by the end point parameter of the fitting of the data to a mono exponential equation using ProFit software (QuantumSoft). For pr titrations, we normalized all the curves to the % of lipid mixing measured in absence of pr. The concentration of viral E protein in the final volume of the assay was quantified by western blot. Shortly, a range of 25 to 200 ng of recombinant E was used as a standard curve and in the same SDS PAGE gel we loaded 0.15 µl to 10 µL of purified virus. The western blot was revealed with the E21.3 antibody. Bands intensities were calculated in ImageJ software (14) and used to interpolate the amount of E in the virus to a standard curve of purified E protein (25-200 ng) by linear regression. The concentration of viral E of 50-80 nM was used to refer the titrated concentrations of pr as a pr/E molar ratio.

Isothermal titration calorimetry (ITC). We titrated 10 µM of E in the cell with several injections of 100 µM pr. The injection volume was 2 µL. We continued the injections beyond saturation to determine the heat of ligand dilution, which was subtracted from the data prior to fitting with a single site binding model. We used Microcal ITC200 from Microcal and the associated Origin software for fitting of the data. The two-component buffer was prepared by dissolving appropriate weights of each component in water (Ellis). The resulting solution had a pH of 8.3, which was taken to the desired value with concentrated HCl. The pH was measured in a Sartorius PB11 pH-meter.

Surface plasmon resonance (SPR). The carboxylic groups of a Series S CM5 sensor chip were activated for 10 min using a mix of N-Hydroxysuccinimide (NHS, 50 mM) and 1-ethyl-3-[3-(dimethylamino)propy1]-carbodiimide (EDC, 200 mM). The Strep-Tactin XT (IBA lifesciences) at 2 µg/mL in acetate pH 5 was injected for 20 min, followed by deactivation with 1 M ethanolamine for 7 min, reaching a density of 800 resonance units (1 RU corresponds to about 1 pg/mm2) of amine coupled Strep-Tactin XT. At the start of each cycle, double strep tagged sE protein was captured on a Strep-Tactin XT surface for 3 min at 5 µg/mL. Eight concentrations of pr peptide (2-fold dilutions ranging from 100 nM to 0.78 nM) were then injected at 30 µl/min for 600s. At the end of each cycle, the surfaces were regenerated by sequential 15s injections of Gly-HCl pH 1.5 and 10 mM NaOH. Experiments were performed in duplicate, using 3 different running buffers, 50 mM MES, 50 mM Tris-HCl (pH 6, 7 and 8) with 150 mM NaCl and 0.2 mg/mL BSA at 25°C (15) (Table 1). The association and dissociation profiles were fitted globally using the Biacore T200 evaluation software (GE Healthcare) assuming a 1:1 interaction between sE and pr.

Virus stocks. We used YF17D-204 genome under a SP6 promoter. In vitro transcribed RNA was electroporated in SW13 cells as previously described (Amberg). Briefly, 3 µg of RNA were mixed with 4 × 10^6 SW-13 cells in PBS and pulsed in 2-mm-gap electroporation cuvettes (BTX) with an electroporator (BTX Electro Square Porator model T820) set for 3 pulses at 800 V with a pulse length of 60 µs. After a 10-min recovery phase at room temperature, cells were

plated in a p75 flask in complete medium (Minimum Essential Medium supplemented with 10% fetal bovine serum (FBS), 1 mM sodium pyruvate, 2 mM Glutamax and 0.1 mM MEM non-essential amino acids). Virus stocks were harvested 48h post-transfection with typical yields of 10^7-10^8 FFU/mL as determined by focus forming assay on SW13. Single use aliquots were stored frozen at -80°C until use.

Virus purification. SW13 cell monolayers were infected at low MOI (0.1 ffu/cell) and supernatants were collected 48h post-infection. YF17D virus was recovered by precipitation with 8%(w/v) PEG 8000 for 1h at 4ºC and purified on a step tartrate-glycerol gradient (40-10%(w/v) tartrate - 5-30%(w/v) glycerol) by over-night ultracentrifugation in SW41 at 30 Krpm. Virus band was recovered by needle puncture at the side of the tube and virus titers were determined by focus forming assay. The total amount of virus present in the preparation was quantified by comparison against known amount of purified sE protein and western blot with YF E-specific antibody E21.3. The virus band buffer corresponded to about 25%(w/v) tartrate, 15%(w/v) glycerol and 0.02%(w/v) BSA. The virus preparation was kept at 4°C until use. Buffer-alone gradients were run and collected in parallel to each virus preparation to be used as blank in the functional assays.

Focus-forming assay. Serial dilutions of the virus preparations (1/10) were prepared in 1% FBS / PBS. Each dilution was added to SW13 cells and foci were developed in the presence of 1,5% methylcellulose for 2 days in 96 well plates. Foci development was stopped by fixation with 4% formaldehyde and foci were then stained using a mouse-anti-NS1 antibody (1A5) (gift from Jacob Schlesinger, Rochester University) and a horseradish peroxidase (HRP) conjugated secondary anti-mouse antibody (ThermoFisher 31430). The foci were visualized by diaminobenzidine (DAB) (Sigma D5905) staining and imaged using the ImmunoSpot S6 Analyser (Cellular Technology Limited).

REFERENCES

1. Iwaki T, Figuera M, Ploplis VA, Castellino FJ. 2003. Rapid selection of *Drosophila* S2 cells with the puromycin resistance gene. BioTechniques 35:482–486.

2. Weber P, Pissis C, Navaza R, Mechaly AE, Saul F, Alzari PM, Haouz A. 2019. High-Throughput Crystallization Pipeline at the Crystallography Core Facility of the Institut Pasteur. Molecules 24:4451.

3. Trapani S, Navaza J. 2008. *AMoRe*: classical and modern. Acta Crystallogr D Biol Crystallogr 64:11–16.

4. Rey FA, Heinz FX, Mandl C, Kunz C, Harrison SC. 1995. The envelope glycoprotein from tick-borne encephalitis virus at 2 Å resolution. Nature 375:291–298.

5. Li L, Lok S-M, Yu I-M, Zhang Y, Kuhn RJ, Chen J, Rossmann MG. 2008. The Flavivirus Precursor Membrane-Envelope Protein Complex: Structure and Maturation. Science 319:1830–1834.

6. Emsley P, Lohkamp B, Scott WG, Cowtan K. 2010. Features and development of *Coot*. Acta Crystallogr D Biol Crystallogr 66:486–501.

7. Bricogne. Bricogne G., Blanc E., Brandl M., Flensburg C., Keller P., Paciorek W., Roversi P, Sharff A., Smart O.S., Vonrhein C., Womack T.O. (2017). BUSTER version 2.10.3. Cambridge, United Kingdom: Global Phasing Ltd.

8. Blanc E, Roversi P, Vonrhein C, Flensburg C, Lea SM, Bricogne G. 2004. Refinement of severely incomplete structures with maximum likelihood in *BUSTER–TNT*. Acta Crystallogr D Biol Crystallogr 60:2210–2221.

9. Afonine PV, Grosse-Kunstleve RW, Echols N, Headd JJ, Moriarty NW, Mustyakimov M, Terwilliger TC, Urzhumtsev A, Zwart PH, Adams PD. 2012. Towards automated crystallographic structure refinement with *phenix.refine*. Acta Crystallogr D Biol Crystallogr 68:352–367.

10. Smart OS, Womack TO, Flensburg C, Keller P, Paciorek W, Sharff A, Vonrhein C, Bricogne G. 2012. Exploiting structure similarity in refinement: automated NCS and target-structure restraints in *BUSTER*. Acta Crystallogr D Biol Crystallogr 68:368–380.

11. Winn MD, Murshudov GN, Papiz MZ. 2003. Macromolecular TLS Refinement in REFMAC at Moderate Resolutions, p. 300–321. *In* Methods in Enzymology. Elsevier.

12. Tickle. Tickle, I.J., Flensburg, C., Keller, P., Paciorek, W., Sharff, A., Vonrhein, C., Bricogne, G. (2018). STARANISO. Cambridge, United Kingdom: Global Phasing Ltd. (http://staraniso.globalphasing.org).

13. Chen VB, Arendall WB, Headd JJ, Keedy DA, Immormino RM, Kapral GJ, Murray LW, Richardson JS, Richardson DC. 2010. *MolProbity*: all-atom structure validation for macromolecular crystallography. Acta Crystallogr D Biol Crystallogr 66:12–21.

14. Schneider CA, Rasband WS, Eliceiri KW. 2012. NIH Image to ImageJ: 25 years of image analysis. Nat Methods 9:671–675.

15. Ellis KJ, Morrison JF. 1982. [23] Buffers of constant ionic strength for studying pH-dependent processes, p. 405–426. *In* Methods in Enzymology. Elsevier.

FIG S1

A

B

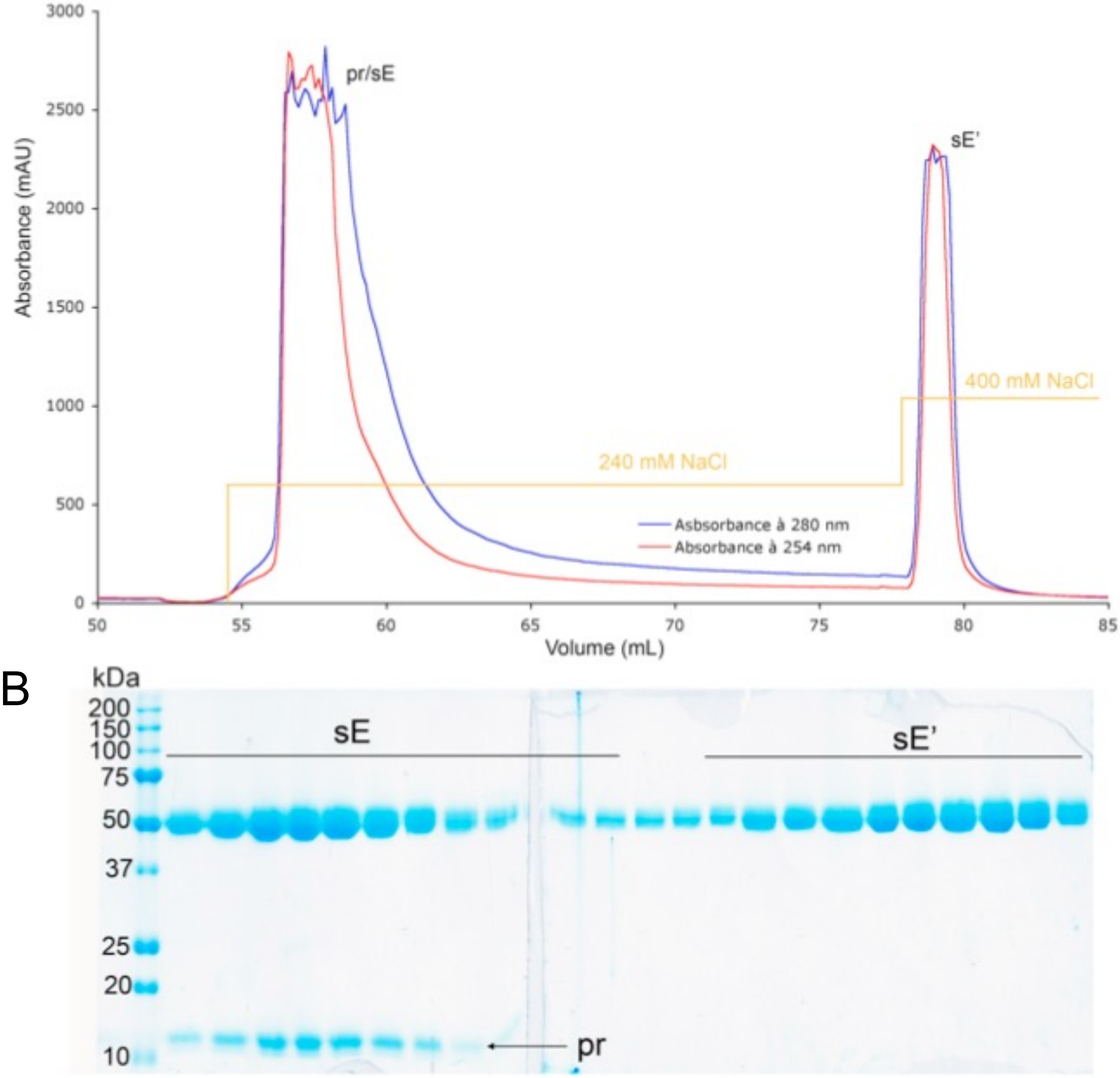

Figure S1. Anion exchange purification of YFV pr/sE complex (related to Material & Methods).

(A) SEC purified YFV pr/E protein was separated on a MonoQ 5/15 with a NaCl step gradient (240mM-400mM).

(B) SDS-PAGE of fractions corresponding to the two peaks purified by Mono-Q column. Peak eluted at 240mM NaCl contains both sE and pr proteins. Peak eluted at 400mM NaCl contains only sE protein.

FIG S2

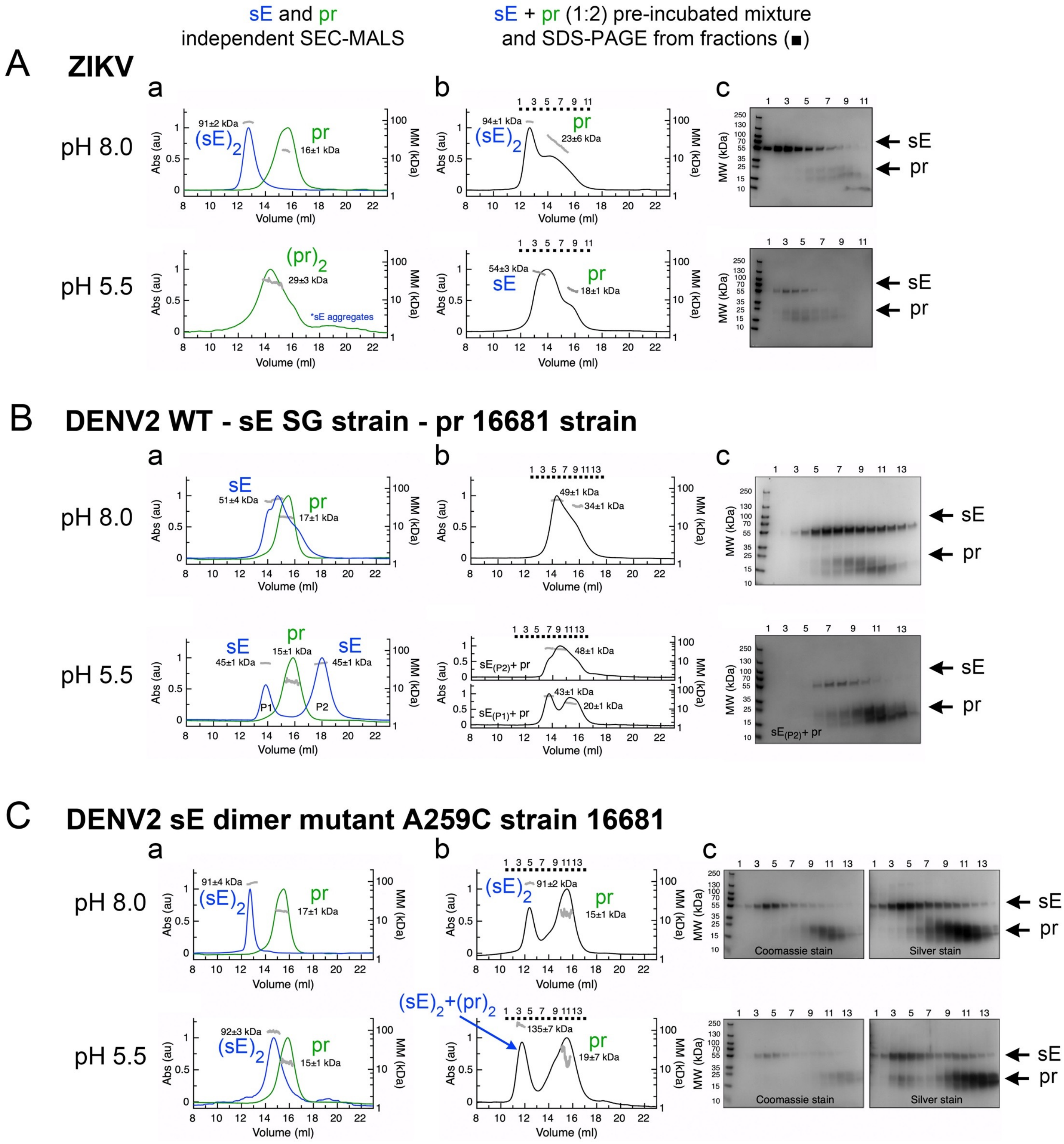

sE and pr
independent SEC-MALS
sE + pr (1:2) pre-incubated mixture
and SDS-PAGE from fractions (■)
A ZIKV
a
b
c
pH 8.0
pH 5.5
(sE)2
pr
91±2 kDa
16±1 kDa
94±1 kDa
23±6 kDa
(pr)2
29±3 kDa
*sE aggregates
54±3 kDa
sE
18±1 kDa
B DENV2 WT - sE SG strain - pr 16681 strain
51±4 kDa
17±1 kDa
49±1 kDa
34±1 kDa
45±1 kDa
15±1 kDa
P1
P2
sE(P2)+ pr
48±1 kDa
sE(P1)+ pr
43±1 kDa
20±1 kDa
C DENV2 sE dimer mutant A259C strain 16681
91±4 kDa
17±1 kDa
91±2 kDa
15±1 kDa
Coomassie stain
Silver stain
92±3 kDa
15±1 kDa
(sE)2+(pr)2
135±7 kDa
19±7 kDa
Abs (au)
MM (kDa)
MW (kDa)
Volume (ml)

FIG S2. SEC-MALS elution volume profiles and SDS-PAGE of isolated sE and pr and of mixture of sE+pr at pH 8.0 and at pH 5.5 for ZIKV, DENV2 WT and DENV2 A259C mutant (related to Fig. 3).

(A, B, C) For each virus, SEC-MALS elution volume profiles of isolated sE, pr (left panels) and incubated pr:sE proteins (middle panels). SDS-PAGE of the fractions indicated on SEC-MALS profiles are on the right panels. SEC-MALS profiles: left y axis, ultraviolet absorbance normalized by setting the highest peak to 1; right y axis, molecular mass (kDa) determined by MALS, with the values for each species indicated on the corresponding peak. **Left panels**: for each virus, SEC-MALS elution volume profiles of isolated sE (blue curves) and isolated pr (green curves) at pH 8.0 (top) and at pH 5.5 (bottom). **Middle panels**: for each virus, SEC-MALS elution volume profiles of a mixture of sE with an excess of pr (1:2 sE:pr monomer:monomer molar ratio) equilibrated at pH 8.0 (top) and at pH 5.5 (bottom). The fractions extracted for analysis by SDS-PAGE are indicated on top of curves. **Right panels**: for each virus, SDS-PAGE of the SEC-MALS fractions indicated on the middle panels at pH 8.0 (top) and at pH 5.5 (bottom). Coomassie blue staining. Only silver nitrate staining is shown for DENV2 sE A259C mutant at both pHs.

FIG S3

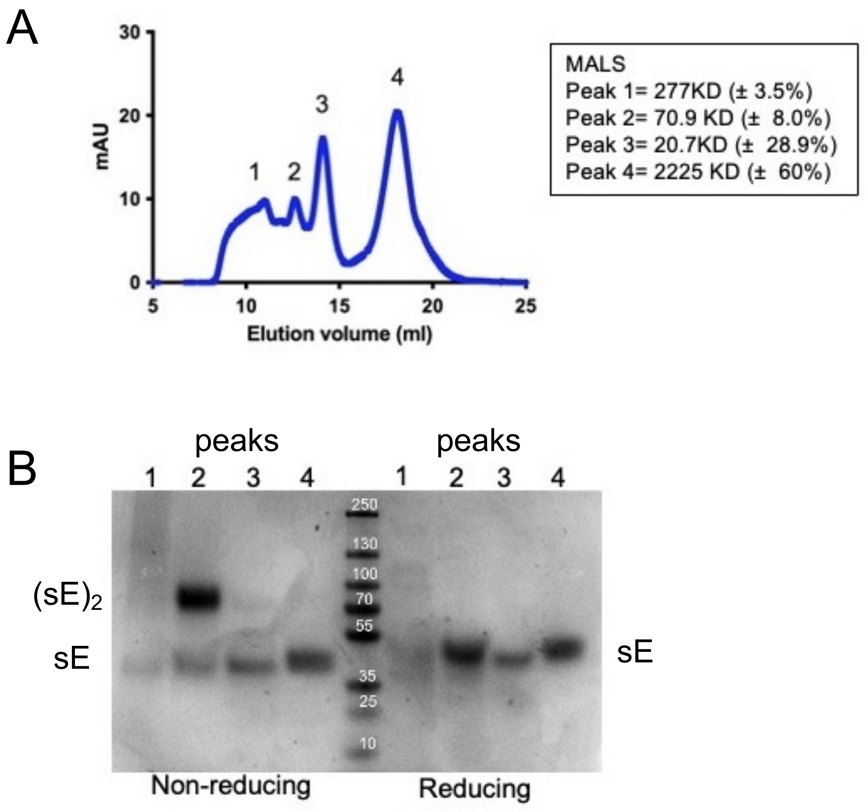

FIG S3. SEC-MALS elution volume profiles and SDS-PAGE of YFV sE S253C mutant (related to Fig. 3).

(A) SEC profile of YFV sE S263C protein. The molecular weight of the four peaks has been measured using MALS and are reported in the inset.

(B) SDS-PAGE of fractions corresponding to the four peaks purified by SEC under non-reducing and reducing condition showing that peak number 2 contains the dimeric from of the protein. Coomassie blue staining.

FIG S4

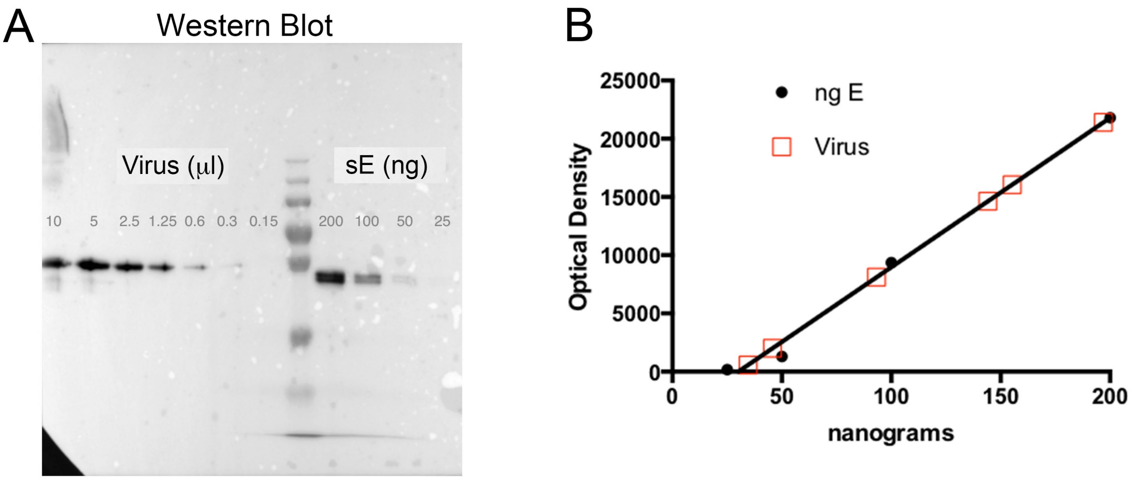

FIG S4. Quantification of E protein on YFV virus.

(A) Western blot with an anti-E specific antibody of serial dilution of purified YFV 17D virus used for the fusion assays. The optical densities of the signal from the virus dilutions and known amounts of purified sE protein has been calculated using Image J software.
(B) The amount of viral E protein has been then calculated by interpolation with the standard curve constructed with the values of the purified E protein using Prism 9.4 software.

TABLE S1 Data collection and refinement statistics.

	Asibi pr/sE	Asibi sE dimer	
**PDB code**	6EPK	8OFN	
Crystallization conditions			
	1.2-1.8M Li_2SO_4<br>0.1M Tris pH 8	1.26M $(NH_4)_2SO_4$<br>0.1M HEPES pH 7.5	
Data Collection [a]			
Synchrotron	SOLEIL	SOLEIL	
Beamline	Proxima-1	Proxima-1	
Space group	P 4_1	P 4_3 2_1 2	
Cell dimensions			
a, b, c (Å)	99.9, 99.9, 238.8	92.5, 92.5, 308.4	
α, β, γ (°)	90, 90, 90	90, 90, 90	
		**AIMLESS**	**STARANISO**
Resolution (Å)	30-2.7 (2.77-2.7)	46.3-3.5 (3.83-3.5)	46.3-3.48 (3.81-3.48) [b]
R*merge*	0.090 (1.123)	0.15 (15.9)	0.12 (2.20)
R*meas*	0.111 (1.394)	0.16 (17.1)	0.12 (2.28)
R*pim*	0.063 (0.814)	0.06 (6.2)	0.03 (0.60)
< *I / σ(I)* >	17.0 (1.2)	8 (0.3)	13.1 (1.5)
$CC_{1/2}$	0.997 (0.52)	0.999 (0.27)	0.999 (0.66)
No. of Unique reflections	63786 (4469)	17868 (4152)	11358 (568)
Multiplicity	5.4 (5.1)	13.3 (13.9)	15.5 (13.8)
Completeness (%)			
Spherical	99.9 (100.0)	99.9 (99.6)	62.8 (13.6)
Ellipsoidal [b]		-	*93.4 (81.5)*
Structure determination			
MR search models	1SVB+3C5X	6EPK	
NCS	2	2	
Refinement [a]			
Resolution	30.0-2.7 (2.75-2.70)	30.0-3.48 (3.81-3.48) [c]	
No. of Work/Free refl.	63742/3182 (2962/160)	11613/983 (664/46)	
Rwork/Rfree	0.16/0.18 (0.34/0.37)	0.25/0.27 (0.29/0.39)	
Targeting	/	6EPK	
No. of atoms/waters	7898/205	5964/0	
Rms deviations from ideality			
Bond lengths (Å)	0.003	0.006	
Bond angles (°)	0.672	0.86	
Ramachandran Plot [d]			
Favored (%)	98.03	97.4	
Allowed (%)	1.97	2.08	
Outliers (%)	0.0	0.52	
Rotamers Outliers	0.61	1.08	

[a] Highest resolution shell is shown in parenthesis.
[b] Data collection statistics were computed after anisotropy correction by STARANISO.
[c] Refinement was done using corrected anisotropy amplitudes.
[d] Ramachandran statistics were calculated with MolProbity.
PDB, Protein Data Bank; NCS, non-crystallographic symmetry; MR, molecular replacement; Rms, root mean square; $CC_{1/2}$, correlation coefficient; Rmeas, multiplicity-corrected R; Rpim, expected precision.

TABLE S2 pr/sE polar interactions in YFV, DENV2 and TBEV.

sE domains			YFV Asibi pr/sE PDB 6EPK (2 HD in au) sE	pr	dist (Å) HD1	HD2	DENV-2 prMe-sE PDB 3C5X (pH 5.5) & 3C6E (pH 7) sE	pr	dist (Å) 3C5X	3C6E	TBEV (pr/sE) dimer PDB 7QRE sE	pr	dist (Å)	pr
Reference subunit E	domain II	b-strand					**LYS 64 [NZ]**	**GLU 46 [OE2]**	**2.5**	**2.9**	**LYS 64 [NZ]**	**ASP 43 [OD2]**	**3.2**	4-strand
							**LYS 64 [NZ]**	**ASP 47 [OD1]**		**4.0**				
			HIS 67 [N]	PRO 42 [O]	2.7	2.6	ASN 67 [N]	GLU 46 [O]		3.9	ASP 67 [N]	ASP 43 [O]	3.1	
			HIS 67 [ND1]	SER 44 [OG]		3.3	ASN 67 [OD1]	THR 48 [OG1]		2.6	ASP 67 [OD1]	SER 45 [OG]	3.6*	
			VAL 68 [N]	SER 44 [O]	3.2	3.2	THR 68 [N]	THR 48 [O]	3.4	3.5	THR 68 [N]	SER 45 [O]	3.3	
											THR 68 [OG1]	SER 45 [O]	3.1	
			VAL 68 [O]	GLU 46 [N]	2.7	2.8	THR 68 [O]	THR 50 [N]	2.7	3.0	THR 68 [O]	SER 47 [N]	2.8	
			ILE 70 [N]	GLU 46 [O]	2.8	2.8	THR 70 [N]	THR 50 [O]	2.9	3.0	VAL 70 [N]	SER 47 [O]	2.9	
			ILE 70 [O]	ARG 72 [NH2]	2.3	2.3								
			ASN 71 [OD1]	ASN 48 [ND2]	3.0	2.8								
			ASP 72 [O]	ASN 48 [ND2]	3.7*	3.7*					ALA 72 [N]	GLU 49 [OE1]	3.1	
			ASP 72 [OD1]	ASN 48 [ND2]	2.6	2.8								
		η1	No interaction				**GLU 84 [OE2]**	**ARG 6 [NH2]**	**3.9**	**3.0**	No interaction			*0-1* loop
		fusion loop (FL)	GLY 102 [O]	ASP 59 [N]	3.0	2.8	GLY 102 [O]	GLU 62 [N]	2.9	2.92	GLY 102 [O]	VAL 60 [N]	2.8	*4-X* loop
			GLY 102 [N]	GLU 56 [OE1]	2.4	2.5								
			ASN 103 [N]	GLU 56 [OE1]	3.2	3.3								
			ASN 103 [ND2]	ASP 59 [O]	3.3	3.6*	ASN 103 [ND2]	GLU 62 [O]	3.0		ASN 103 [ND2]	VAL 60 [O]	3.3	
							ASN 103 [ND2]	GLU 62 [OE1]	3.9*					
			GLY 104 [N]	GLU 56 [OE1]	2.7	2.7					**HIS 104 [ND1]**	**ASP 54 [OD1]**	**3.4**	
											HIS 104 [ND1]	THR 52 [O]	2.8	
		ij-loop	**HIS 238 [ND1]**	**ASP 60 [OD1]**	**2.7**	**3.0**	**HIS 244 [ND1]**	**ASP 63 [OD1]**	**3.5**	**2.7**	**HIS 248 [ND1]**	**ASP 61 [OD1]**	**2.8**	*4-X* loop
			**HIS 238 [ND1]**	**ASP 60 [OD2]**	**3.1**	**3.3**	**HIS 244 [ND1]**	**ASP 63 [OD2]**	**2.8**	**3.6**				
			ALA 239 [N]	ASP 60 [O]	3.5	3.5	ALA 245 [N]	ASP 63 [O]	3.7*	3.8*	ALA 249 [N]	ASP 61 [O]	3.7*	
			ALA 239 [N]	ASP 60 [OD2]	3.2	3.1	ALA 245 [N]	ASP 63 [OD1]	3.0	2.8	ALA 249 [N]	ASP 61 [OD2]	3.2	
							**LYS 247 [NZ]**	**ASP 65 [OD1]**	**2.9**	**2.9**	**LYS 251 [NZ]**	**ASP 63 [OD2]**	**2.8**	
							**LYS 247 [NZ]**	**ASP 65 [OD2]**	**3.8**	**3.7**				
			**ARG 243 [NH2]**	**GLU 36 [OE2]**	**3.8**	**2.4**	**LYS 247 [NZ]**	**ASP 40 [OD1]**	**3.9**	**4.0**	**LYS 251 [NZ]**	**ASP 37 [OD1]**	**3.9**	*3-4* loop
			**ARG 243 [NE]**	**GLU 36 [OE2]**	**3.5**									
			No interaction				LYS 247 [NZ]	TYR 77 [OH]	3.0	2.9	LYS 251 [NZ]	TYR 76 [OH]	3.3	*5*-strand
							LYS 247 [NZ]	TYR 51 [OH]		2.9				
											**ASP 253 [OD1]**	**ARG 78 [NH1]**	**3.1**	
											**ASP 253 [OD2]**	**ARG 78 [NH1]**	**3.9**	
Opposite subunit E	domain I	N-ter	NA				NA				ARG 2 [N]	GLU 58 [OE1]	3.5	*4-X* loop
		150-loop	NA				NA				**GLU 155 [OE1]**	**ARG 67 [NE]**	**2.9**	*X* strand
											**GLU 155 [OE2]**	**ARG 67 [NE]**	**3.4**	
											**GLU 155 [OE2]**	**ARG 67 [NH2]**	**3.0**	
											**HIS 157 [ND1]**	**GLU 58 [OE1]**	**3.6**	*4-X* loop
											**HIS 157 [ND1]**	**GLU 58 [OE2]**	**3.2**	
		E0-F0 loop	NA				NA				**ARG 160 [NH2]**	**GLU 58 [OE1]**	**3.4**	
	domain III	A-strand	NA				NA				LYS 315 [NZ]	GLN 55 [O]	3.7*	*4-X* loop
											LYS 315 [NZ]	GLY 56 [O]	3.9*	
											**ARG 316 [NH2]**	**ASP 54 [OD1]**	**3.2**	
											**ARG 316 [NH1]**	**GLU 57 [OE2]**	**3.4**	
											**GLU 329 [OE1]**	**GLU 57 [OE2]**	**3.1**	
											**GLU 329 [OE2]**	**GLU 57 [OE2]**	**3.0**	

Polar contacts are computed with PDBePISA 'Protein interfaces, surfaces and assemblies'
Conserved interactions are on green background.
In red: main chain atoms involved in H-bonds; In bold black: salt bridges; In blue and bold: acidic interactions.
Hydrogen bonds distances cut-off: 3.5Å; Salt bridges distances cut-off: 4Å. * indicates H-bonds weaker with distances between 3.5Å and 4.1Å
NA: non applicable; HD: heterodimer; au: asymetric unit

TABLE S3 pr/sE van der Waals interactions in YFV, DENV2 and TBEV.

<table>
<tr><th colspan="6">YFV (PDB 6EPK)</th></tr>
<tr><th>Res.</th><th>ASA (Å)</th><th>BSA (Å)</th><th></th><th>BSA / ASA (%)</th><th>BSA / BSA total (%)</th></tr>
<tr><td>sE</td><td></td><td></td><td></td><td></td><td></td></tr>
<tr><td>LEU 65</td><td>28.7</td><td>13.7</td><td>|||||</td><td>48.0</td><td>1.7</td></tr>
<tr><td>THR 66</td><td>54.1</td><td>33.3</td><td>|||||||</td><td>61.6</td><td>4.0</td></tr>
<tr><td>HIS 67</td><td>134.9</td><td>67.4</td><td>|||||</td><td>50.0</td><td>8.2</td></tr>
<tr><td>VAL 68</td><td>63.5</td><td>58.3</td><td>||||||||||</td><td>91.8</td><td>7.1</td></tr>
<tr><td>LYS 69</td><td>92.7</td><td>41.8</td><td>|||||</td><td>45.1</td><td>5.1</td></tr>
<tr><td>ILE 70</td><td>52.0</td><td>52.0</td><td>||||||||||</td><td>100.0</td><td>6.3</td></tr>
<tr><td>ASN 71</td><td>49.3</td><td>30.5</td><td>|||||||</td><td>61.9</td><td>3.7</td></tr>
<tr><td>ASP 72</td><td>41.8</td><td>21.1</td><td>||||||</td><td>50.4</td><td>2.6</td></tr>
<tr><td>LEU 82</td><td>33.0</td><td>8.4</td><td>|||</td><td>25.4</td><td>1.0</td></tr>
<tr><td>ASP 98</td><td>97.8</td><td>1.9</td><td>|</td><td>1.9</td><td>0.2</td></tr>
<tr><td>ARG 99</td><td>19.5</td><td>5.5</td><td>|||</td><td>28.2</td><td>0.7</td></tr>
<tr><td>TRP 101</td><td>221.6</td><td>91.6</td><td>|||||</td><td>41.3</td><td>11.1</td></tr>
<tr><td>GLY 102</td><td>73.1</td><td>71.9</td><td>||||||||||</td><td>98.4</td><td>8.7</td></tr>
<tr><td>ASN 103</td><td>56.8</td><td>49.3</td><td>|||||||||</td><td>86.7</td><td>6.0</td></tr>
<tr><td>GLY 104</td><td>73.2</td><td>44.1</td><td>|||||||</td><td>60.2</td><td>5.3</td></tr>
<tr><td>CYS 105</td><td>4.7</td><td>0.2</td><td>|</td><td>3.2</td><td>0.0</td></tr>
<tr><td>PRO 237</td><td>42.8</td><td>0.9</td><td>|</td><td>2.0</td><td>0.1</td></tr>
<tr><td>HIS 238</td><td>127.8</td><td>34.8</td><td>|||</td><td>27.2</td><td>4.2</td></tr>
<tr><td>ALA 239</td><td>27.8</td><td>27.5</td><td>||||||||||</td><td>99.0</td><td>3.3</td></tr>
<tr><td>ALA 240</td><td>54.8</td><td>52.0</td><td>||||||||||</td><td>94.9</td><td>6.3</td></tr>
<tr><td>THR 241</td><td>70.8</td><td>46.3</td><td>|||||||</td><td>65.3</td><td>5.6</td></tr>
<tr><td>ILE 242</td><td>7.1</td><td>5.2</td><td>||||||||</td><td>72.4</td><td>0.6</td></tr>
<tr><td>ARG 243</td><td>155.0</td><td>37.5</td><td>|||</td><td>24.2</td><td>4.5</td></tr>
<tr><td>VAL 244</td><td>15.2</td><td>10.7</td><td>||||||||</td><td>70.3</td><td>1.3</td></tr>
<tr><td>LEU 245</td><td>94.0</td><td>17.2</td><td>||</td><td>18.3</td><td>2.1</td></tr>
<tr><td>pr</td><td></td><td></td><td></td><td></td><td></td></tr>
<tr><td>TRP 9</td><td>85.4</td><td>11.7</td><td>||</td><td>13.7</td><td>1.4</td></tr>
<tr><td>GLU 36</td><td>84.1</td><td>39.0</td><td>|||||</td><td>46.4</td><td>4.7</td></tr>
<tr><td>TRP 40</td><td>148.7</td><td>10.8</td><td>|</td><td>7.3</td><td>1.3</td></tr>
<tr><td>PRO 42</td><td>139.0</td><td>62.6</td><td>|||||</td><td>45.0</td><td>7.6</td></tr>
<tr><td>ASP 43</td><td>77.8</td><td>22.6</td><td>|||</td><td>29.1</td><td>2.7</td></tr>
<tr><td>SER 44</td><td>43.8</td><td>40.4</td><td>||||||||||</td><td>92.1</td><td>4.9</td></tr>
<tr><td>MET 45</td><td>73.7</td><td>69.2</td><td>||||||||||</td><td>93.9</td><td>8.4</td></tr>
<tr><td>GLU 46</td><td>89.7</td><td>57.7</td><td>|||||||</td><td>64.3</td><td>7.0</td></tr>
<tr><td>TYR 47</td><td>52.3</td><td>49.3</td><td>||||||||||</td><td>94.2</td><td>6.0</td></tr>
<tr><td>ASN 48</td><td>81.9</td><td>58.9</td><td>||||||||</td><td>71.9</td><td>7.1</td></tr>
<tr><td>PRO 50</td><td>51.5</td><td>32.5</td><td>|||||||</td><td>63.1</td><td>3.9</td></tr>
<tr><td>ASN 51</td><td>61.7</td><td>13.7</td><td>|||</td><td>22.2</td><td>1.7</td></tr>
<tr><td>LEU 52</td><td>17.5</td><td>10.5</td><td>|||||||</td><td>60.0</td><td>1.3</td></tr>
<tr><td>SER 53</td><td>63.3</td><td>21.4</td><td>||||</td><td>33.8</td><td>2.6</td></tr>
<tr><td>ARG 55</td><td>228.3</td><td>15.1</td><td>|</td><td>6.6</td><td>1.8</td></tr>
<tr><td>GLU 56</td><td>74.8</td><td>71.3</td><td>||||||||||</td><td>95.4</td><td>8.6</td></tr>
<tr><td>GLU 57</td><td>144.6</td><td>10.8</td><td>|</td><td>7.5</td><td>1.3</td></tr>
<tr><td>PRO 58</td><td>49.1</td><td>0.5</td><td>|</td><td>1.0</td><td>0.1</td></tr>
<tr><td>ASP 59</td><td>116.0</td><td>39.9</td><td>||||</td><td>34.4</td><td>4.8</td></tr>
<tr><td>ASP 60</td><td>149.0</td><td>77.0</td><td>||||||</td><td>51.7</td><td>9.3</td></tr>
<tr><td>ILE 61</td><td>54.6</td><td>31.6</td><td>||||||</td><td>57.9</td><td>3.8</td></tr>
<tr><td>ASP 62</td><td>51.0</td><td>12.0</td><td>|||</td><td>23.6</td><td>1.5</td></tr>
<tr><td>ARG 72</td><td>56.5</td><td>40.4</td><td>||||||||</td><td>71.6</td><td>4.9</td></tr>
<tr><td>LYS 77</td><td>63.7</td><td>24.6</td><td>||||</td><td>38.7</td><td>3.0</td></tr>
</table>

<table>
<tr><th colspan="6">DENV2 (PDB 3C5X)</th></tr>
<tr><th>Res.</th><th>ASA (Å)</th><th>BSA (Å)</th><th></th><th>BSA / ASA (%)</th><th>BSA / BSA total (%)</th></tr>
<tr><td>sE</td><td></td><td></td><td></td><td></td><td></td></tr>
<tr><td>LYS 64</td><td>100.7</td><td>40.7</td><td>|||||</td><td>40.5</td><td>5.1</td></tr>
<tr><td>LEU 65</td><td>30.1</td><td>10.6</td><td>||||</td><td>35.1</td><td>1.3</td></tr>
<tr><td>THR 66</td><td>49.2</td><td>33.5</td><td>|||||||</td><td>68.1</td><td>4.2</td></tr>
<tr><td>ASN 67</td><td>80.3</td><td>19.4</td><td>|||</td><td>24.2</td><td>2.4</td></tr>
<tr><td>THR 68</td><td>72.4</td><td>56.1</td><td>||||||||</td><td>77.5</td><td>7.0</td></tr>
<tr><td>THR 69</td><td>50.3</td><td>41.9</td><td>|||||||||</td><td>83.3</td><td>5.2</td></tr>
<tr><td>THR 70</td><td>33.7</td><td>30.0</td><td>|||||||||</td><td>89.1</td><td>3.7</td></tr>
<tr><td>GLU 71</td><td>63.0</td><td>29.7</td><td>|||||</td><td>47.1</td><td>3.7</td></tr>
<tr><td>LEU 82</td><td>27.6</td><td>14.7</td><td>||||||</td><td>53.2</td><td>1.8</td></tr>
<tr><td>ASN 83</td><td>136.4</td><td>21.9</td><td>||</td><td>16.1</td><td>2.7</td></tr>
<tr><td>GLU 84</td><td>45.8</td><td>15.5</td><td>||||</td><td>33.8</td><td>1.9</td></tr>
<tr><td>VAL 97</td><td>12.7</td><td>2.3</td><td>||</td><td>18.4</td><td>0.3</td></tr>
<tr><td>ASP 98</td><td>93.4</td><td>3.0</td><td>|</td><td>3.2</td><td>0.4</td></tr>
<tr><td>ARG 99</td><td>9.5</td><td>0.6</td><td>|</td><td>6.1</td><td>0.1</td></tr>
<tr><td>TRP 101</td><td>183.6</td><td>48.4</td><td>|||</td><td>26.3</td><td>6.0</td></tr>
<tr><td>GLY 102</td><td>79.2</td><td>71.5</td><td>||||||||||</td><td>90.3</td><td>8.9</td></tr>
<tr><td>ASN 103</td><td>52.8</td><td>50.9</td><td>||||||||||</td><td>96.4</td><td>6.3</td></tr>
<tr><td>GLY 104</td><td>70.2</td><td>31.8</td><td>|||||</td><td>45.3</td><td>3.9</td></tr>
<tr><td>PHE 108</td><td>135.0</td><td>12.7</td><td>|</td><td>9.4</td><td>1.6</td></tr>
<tr><td>ARG 120</td><td>154.9</td><td>11.5</td><td>|</td><td>7.4</td><td>1.4</td></tr>
<tr><td>LYS 122</td><td>85.5</td><td>0.9</td><td>|</td><td>1.1</td><td>0.1</td></tr>
<tr><td>PRO 243</td><td>29.4</td><td>0.2</td><td>|</td><td>0.8</td><td>0.0</td></tr>
<tr><td>HIS 244</td><td>114.2</td><td>32.9</td><td>|||</td><td>28.8</td><td>4.1</td></tr>
<tr><td>ALA 245</td><td>20.4</td><td>20.4</td><td>||||||||||</td><td>100.0</td><td>2.5</td></tr>
<tr><td>LYS 246</td><td>108.2</td><td>92.1</td><td>|||||||||</td><td>85.1</td><td>11.4</td></tr>
<tr><td>LYS 247</td><td>128.5</td><td>102.8</td><td>||||||||</td><td>80.0</td><td>12.8</td></tr>
<tr><td>ASP 249</td><td>83.0</td><td>10.2</td><td>||</td><td>12.3</td><td>1.3</td></tr>
<tr><td>pr</td><td></td><td></td><td></td><td></td><td></td></tr>
<tr><td>ARG 6</td><td>99.7</td><td>56.7</td><td>||||||</td><td>56.9</td><td>7.0</td></tr>
<tr><td>ASN 7</td><td>134.6</td><td>16.4</td><td>||</td><td>12.2</td><td>2.0</td></tr>
<tr><td>ASP 40</td><td>75.1</td><td>7.7</td><td>||</td><td>10.2</td><td>1.0</td></tr>
<tr><td>GLU 46</td><td>173.7</td><td>71.5</td><td>|||||</td><td>41.2</td><td>8.9</td></tr>
<tr><td>ASP 47</td><td>87.3</td><td>30.2</td><td>||||</td><td>34.6</td><td>3.7</td></tr>
<tr><td>THR 48</td><td>59.9</td><td>32.2</td><td>||||||</td><td>53.7</td><td>4.0</td></tr>
<tr><td>ILE 49</td><td>65.3</td><td>37.8</td><td>||||||</td><td>57.9</td><td>4.7</td></tr>
<tr><td>THR 50</td><td>61.3</td><td>46.0</td><td>||||||||</td><td>75.1</td><td>5.7</td></tr>
<tr><td>TYR 51</td><td>57.9</td><td>53.9</td><td>||||||||||</td><td>93.1</td><td>6.7</td></tr>
<tr><td>LYS 52</td><td>71.3</td><td>39.9</td><td>||||||</td><td>56.0</td><td>4.9</td></tr>
<tr><td>PRO 54</td><td>45.7</td><td>36.0</td><td>||||||||</td><td>78.8</td><td>4.5</td></tr>
<tr><td>LEU 55</td><td>63.2</td><td>8.6</td><td>||</td><td>13.6</td><td>1.1</td></tr>
<tr><td>LEU 56</td><td>43.4</td><td>26.4</td><td>|||||||</td><td>60.8</td><td>3.3</td></tr>
<tr><td>ASN 59</td><td>113.3</td><td>44.8</td><td>||||</td><td>39.5</td><td>5.6</td></tr>
<tr><td>GLU 60</td><td>154.0</td><td>8.6</td><td>|</td><td>5.6</td><td>1.1</td></tr>
<tr><td>PRO 61</td><td>53.3</td><td>0.3</td><td>|</td><td>0.6</td><td>0.0</td></tr>
<tr><td>GLU 62</td><td>139.4</td><td>47.9</td><td>||||</td><td>34.3</td><td>5.9</td></tr>
<tr><td>ASP 63</td><td>138.9</td><td>77.3</td><td>||||||</td><td>55.7</td><td>9.6</td></tr>
<tr><td>ILE 64</td><td>67.2</td><td>41.1</td><td>|||||||</td><td>61.2</td><td>5.1</td></tr>
<tr><td>ASP 65</td><td>47.0</td><td>17.5</td><td>||||</td><td>37.1</td><td>2.2</td></tr>
<tr><td>TRP 74</td><td>50.0</td><td>42.4</td><td>|||||||||</td><td>84.8</td><td>5.3</td></tr>
<tr><td>TYR 77</td><td>1.5</td><td>1.5</td><td>||||||||||</td><td>100.0</td><td>0.2</td></tr>
</table>

<table>
<tr><th colspan="6">TBEV (PDB 7QRE)</th></tr>
<tr><th>Res.</th><th>ASA (Å)</th><th>BSA (Å)</th><th></th><th>BSA / ASA (%)</th><th>BSA / BSA total (%)</th></tr>
<tr><td>sE</td><td></td><td></td><td></td><td></td><td></td></tr>
<tr><td>LYS 64</td><td>105.2</td><td>24.2</td><td>|||</td><td>23.0</td><td>3.0</td></tr>
<tr><td>LEU 65</td><td>39.2</td><td>18.4</td><td>|||||</td><td>46.9</td><td>2.3</td></tr>
<tr><td>SER 66</td><td>44.4</td><td>29.7</td><td>|||||||</td><td>66.9</td><td>3.7</td></tr>
<tr><td>ASP 67</td><td>90.8</td><td>41.7</td><td>|||||</td><td>45.9</td><td>5.2</td></tr>
<tr><td>THR 68</td><td>63.3</td><td>60.1</td><td>||||||||||</td><td>94.9</td><td>7.5</td></tr>
<tr><td>LYS 69</td><td>75.7</td><td>19.2</td><td>|||</td><td>25.4</td><td>2.4</td></tr>
<tr><td>VAL 70</td><td>50.2</td><td>45.8</td><td>||||||||||</td><td>91.2</td><td>5.7</td></tr>
<tr><td>ALA 71</td><td>22.4</td><td>5.4</td><td>|||</td><td>23.9</td><td>0.7</td></tr>
<tr><td>ALA 72</td><td>30.1</td><td>19.3</td><td>|||||||</td><td>64.0</td><td>2.4</td></tr>
<tr><td>ASP 98</td><td>101.1</td><td>7.4</td><td>|</td><td>7.3</td><td>0.9</td></tr>
<tr><td>ARG 99</td><td>18.4</td><td>5.7</td><td>||||</td><td>30.9</td><td>0.7</td></tr>
<tr><td>TRP 101</td><td>184.2</td><td>44.2</td><td>|||</td><td>24.0</td><td>5.5</td></tr>
<tr><td>GLY 102</td><td>83.7</td><td>68.7</td><td>|||||||||</td><td>82.1</td><td>8.5</td></tr>
<tr><td>ASN 103</td><td>49.2</td><td>40.1</td><td>|||||||||</td><td>81.4</td><td>5.0</td></tr>
<tr><td>HIS 104</td><td>172.2</td><td>88.5</td><td>||||||</td><td>51.4</td><td>11.0</td></tr>
<tr><td>PRO 247</td><td>39.0</td><td>0.1</td><td>|</td><td>0.3</td><td>0.0</td></tr>
<tr><td>HIS 248</td><td>123.9</td><td>32.4</td><td>|||</td><td>26.2</td><td>4.0</td></tr>
<tr><td>ALA 249</td><td>26.3</td><td>26.3</td><td>||||||||||</td><td>100.0</td><td>3.3</td></tr>
<tr><td>VAL 250</td><td>81.9</td><td>78.0</td><td>||||||||||</td><td>95.3</td><td>9.7</td></tr>
<tr><td>LYS 251</td><td>136.8</td><td>102.2</td><td>||||||||</td><td>74.7</td><td>12.7</td></tr>
<tr><td>MET 252</td><td>2.2</td><td>1.6</td><td>|||||||</td><td>70.1</td><td>0.2</td></tr>
<tr><td>ASP 253</td><td>72.0</td><td>43.4</td><td>|||||||</td><td>60.3</td><td>5.4</td></tr>
<tr><td>VAL 254</td><td>19.5</td><td>8.8</td><td>|||||</td><td>45.1</td><td>1.1</td></tr>
<tr><td>pr</td><td></td><td></td><td></td><td></td><td></td></tr>
<tr><td>ALA 35</td><td>1.5</td><td>0.2</td><td>||</td><td>11.3</td><td>0.0</td></tr>
<tr><td>ASP 37</td><td>81.0</td><td>15.7</td><td>||</td><td>19.4</td><td>1.9</td></tr>
<tr><td>TRP 41</td><td>93.7</td><td>3.1</td><td>|</td><td>3.3</td><td>0.4</td></tr>
<tr><td>CYS 42</td><td>32.9</td><td>0.5</td><td>|</td><td>1.5</td><td>0.1</td></tr>
<tr><td>ASP 43</td><td>150.0</td><td>76.3</td><td>||||||</td><td>50.8</td><td>9.5</td></tr>
<tr><td>ASP 44</td><td>77.9</td><td>18.5</td><td>|||</td><td>23.8</td><td>2.3</td></tr>
<tr><td>SER 45</td><td>46.6</td><td>40.8</td><td>|||||||||</td><td>87.6</td><td>5.1</td></tr>
<tr><td>LEU 46</td><td>76.0</td><td>76.0</td><td>||||||||||</td><td>100.0</td><td>9.4</td></tr>
<tr><td>SER 47</td><td>74.3</td><td>51.5</td><td>|||||||</td><td>69.3</td><td>6.4</td></tr>
<tr><td>TYR 48</td><td>58.3</td><td>57.1</td><td>||||||||||</td><td>97.8</td><td>7.1</td></tr>
<tr><td>GLU 49</td><td>105.5</td><td>73.3</td><td>|||||||</td><td>69.5</td><td>9.1</td></tr>
<tr><td>VAL 51</td><td>45.7</td><td>40.5</td><td>|||||||||</td><td>88.6</td><td>5.0</td></tr>
<tr><td>THR 52</td><td>49.0</td><td>31.2</td><td>|||||||</td><td>63.7</td><td>3.9</td></tr>
<tr><td>ILE 53</td><td>27.3</td><td>25.2</td><td>||||||||||</td><td>92.4</td><td>3.1</td></tr>
<tr><td>ASP 54</td><td>113.5</td><td>24.6</td><td>|||</td><td>21.7</td><td>3.1</td></tr>
<tr><td>GLU 57</td><td>103.0</td><td>46.1</td><td>|||||</td><td>44.7</td><td>5.7</td></tr>
<tr><td>GLU 58</td><td>138.4</td><td>5.6</td><td>|</td><td>4.1</td><td>0.7</td></tr>
<tr><td>PRO 59</td><td>41.3</td><td>0.2</td><td>|</td><td>0.4</td><td>0.0</td></tr>
<tr><td>VAL 60</td><td>113.8</td><td>32.2</td><td>|||</td><td>28.3</td><td>4.0</td></tr>
<tr><td>ASP 61</td><td>152.1</td><td>81.8</td><td>||||||</td><td>53.8</td><td>10.1</td></tr>
<tr><td>VAL 62</td><td>48.0</td><td>29.0</td><td>|||||||</td><td>60.3</td><td>3.6</td></tr>
<tr><td>ASP 63</td><td>53.5</td><td>23.1</td><td>|||||</td><td>43.2</td><td>2.9</td></tr>
<tr><td>CYS 64</td><td>1.7</td><td>0.5</td><td>|||</td><td>28.3</td><td>0.1</td></tr>
<tr><td>ASP 70</td><td>114.7</td><td>10.5</td><td>|</td><td>9.2</td><td>1.3</td></tr>
<tr><td>TYR 73</td><td>85.5</td><td>7.5</td><td>|</td><td>8.8</td><td>0.9</td></tr>
<tr><td>TYR 76</td><td>4.0</td><td>4.0</td><td>||||||||||</td><td>100.0</td><td>0.5</td></tr>
<tr><td>ARG 78</td><td>94.5</td><td>33.6</td><td>||||</td><td>35.6</td><td>4.2</td></tr>
</table>

TABLE S3. pr/sE van der Waals interactions in YFV, DENV2 and TBEV.

The accessible surface area (ASA) and the buried surface area (BSA) were computed with PDBePISA server (https://www.ebi.ac.uk/msd-srv/prot_int/pistart.html). The ASA and BSA were computed for each residue at the interface of pr and sE in the pr/sE complexes of YFV, DENV2 and TBEV. For TBEV, only the pr/sE monomer was used for the computation to compare with the interaction of pr/sE in YFV and DENV2. BSA represent both the polar and the apolar fractions of the surface that are buried upon complexation of pr with E and of E with pr. The vertical bars in the BSA columns reflect how much each residue is buried at the interface pr/sE. The percentage of BSA is computed as ‘BSA/ASA’ for each residue and as ‘BSA/BSA total’, ‘BSA total’ representing the total buried surface area at the interface of pr and E. The ‘BSA total’ at the interface of pr and sE for YFV, DENV2 and TBEV is 825 Å2, 806.1 Å2 and 808.6 Å2, respectively.
